# Deficiency of histone lysine methyltransferase SETDB2 in hematopoietic cells promotes vascular inflammation and accelerates atherosclerosis

**DOI:** 10.1172/jci.insight.147984

**Published:** 2021-06-22

**Authors:** Xinbo Zhang, Jonathan Sun, Alberto Canfrán-Duque, Binod Aryal, George Tellides, Ying Ju Chang, Yajaira Suárez, Timothy F. Osborne, Carlos Fernández-Hernando

**Affiliations:** 1 Vascular Biology and Therapeutics Program,; 2 Integrative Cell Signaling and Neurobiology of Metabolism Program, Department of Comparative Medicine and Department of Pathology, and; 3 Department of Surgery, Yale University School of Medicine, New Haven, Connecticut, USA.; 4 Department of Medicine and; 5 Institute for Fundamental Biomedical Research, Johns Hopkins University School of Medicine, St. Petersburg, Florida, USA.

**Keywords:** Inflammation, Vascular Biology, Atherosclerosis, Macrophages

## Abstract

Epigenetic modifications of the genome, including DNA methylation, histone methylation/acetylation, and noncoding RNAs, have been reported to play a fundamental role in regulating immune response during the progression of atherosclerosis. SETDB2 is a member of the KMT1 family of lysine methyltransferases, and members of this family typically methylate histone H3 Lys9 (H3K9), an epigenetic mark associated with gene silencing. Previous studies have shown that SETDB2 is involved in innate and adaptive immunity, the proinflammatory response, and hepatic lipid metabolism. Here, we report that expression of SETDB2 is markedly upregulated in human and murine atherosclerotic lesions. Upregulation of SETDB2 was observed in proinflammatory M1 but not antiinflammatory M2 macrophages. Notably, we found that genetic deletion of SETDB2 in hematopoietic cells promoted vascular inflammation and enhanced the progression of atherosclerosis in BM transfer studies in *Ldlr-*knockout mice. Single-cell RNA-Seq analysis in isolated CD45^+^ cells from atherosclerotic plaques from mice transplanted with SETDB2-deficient BM revealed a significant increase in monocyte population and enhanced expression of genes involved in inflammation and myeloid cell recruitment. Additionally, we found that loss of SETDB2 in hematopoietic cells was associated with macrophage accumulation in atherosclerotic lesions and attenuated efferocytosis. Overall, these studies identify SETDB2 as an important inflammatory cell regulator that controls macrophage activation in atherosclerotic plaques.

## Graphical abstract

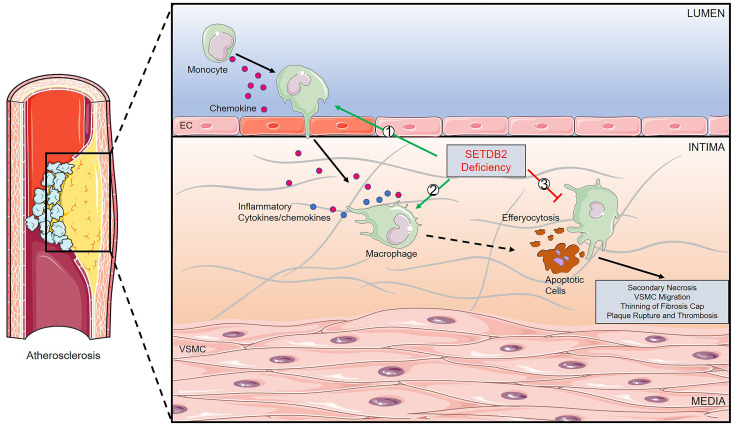

## Introduction

Atherosclerosis is characterized by chronic inflammation and disordered lipid metabolism during the initiation and development of this disease, which leads to acute cardiovascular events, such as myocardial infarction and stroke (1). It starts as an endothelial response to injury that leads to lipid deposition in the vessel wall, followed by circulating monocyte infiltration, mediated by chemokines and proinflammatory cytokines, macrophage foam cell formation, impaired resolution of persistent inflammation, and, eventually, plaque rupture and thrombosis (1, 2). The relevance of epigenetic modifications that regulate gene expression in vascular and immune cells during atherogenesis, including DNA methylation, histone methylation/acetylation, and noncoding RNAs, has been increasingly recognized (3). Deficiency of DNA-demethylating enzyme TET methylcytosine dioxygenase 2 (TET2) in hematopoietic cells accelerates atherosclerosis by elevating expression of cytokines and chemokines and NLRP3 inflammasome activation (4, 5). Histone methyltransferase enhancer of zeste homolog 2 (EZH2) induces DNA methyltransferase 1 (DNMT1) expression and methyl-CpG–binding protein-2 (MeCP2) recruitment to the ATP-binding cassette transporter A1 (*ABCA1*) gene promoter, suppressing cellular cholesterol efflux and promoting foam cell formation and atherogenesis (6).

SETDB2 (su(var) 3-9–enhancer-of-zeste–trithorax [SET] domain bifurcated 2) belongs to the KMT1 family of lysine methyltransferases that includes the SUV39 and SETDB1, which have been shown to bind AdoMet in their SET domain and transfer methyl residues to the amino group of histone H3 Lys9 (H3K9) to silence gene expression (7). SETDB2 has been previously reported to be involved in innate and adaptive immunity, proinflammatory responses, and T cell differentiation through modulation of the expression of NF-κB target genes and type I IFN responses (8, 9). In this setting, SETDB2 loss is associated with a prolonged proinflammatory response, in which a subset of proinflammatory genes are not repressed normally to resolve the initial inflammatory response. The prolonged expression is associated with reduced H3K9 methylation at the promoters of affected genes. Independently, we have shown that SETDB2 is induced during fasting in the liver and functions together with the glucocorticoid receptor (GR) to regulate a subset of GR-target genes, including insulin-induced gene 2 (INSIG2a), which function to inhibit SREBPs that mediate lipid metabolism during fasting (10). Together, the functional relevance of SETDB2 in inflammation, immunity and lipid metabolism suggest that SETDB2 might regulate atherosclerosis.

In this study, we report that SETDB2 expression is induced upon proinflammatory activation in macrophages and is highly expressed in macrophages that accumulate in human and mouse atherosclerotic plaques. We further show that deficiency of SETDB2 in hematopoietic cells promoted the progression of atherosclerosis and plaque instability in atherosclerotic mice. Notably, single-cell RNA-Seq (scRNA-Seq) analysis in isolated CD45^+^ cells from atherosclerotic plaques revealed that SETDB2 controls the recruitment of inflammatory monocytes to atherosclerotic lesions and increases the expression of proinflammatory cytokines and chemokines. SETDB2 deficiency in macrophages promoted an LPS-induced inflammatory response and impaired the engulfment of apoptotic cells in vitro, which may contribute to the molecular mechanisms of SETDB2 in atherogenesis.

## Results

### Macrophage SETDB2 expression is upregulated in response to innate immune activation but it is not affected by cholesterol accumulation.

**Figure 1:**
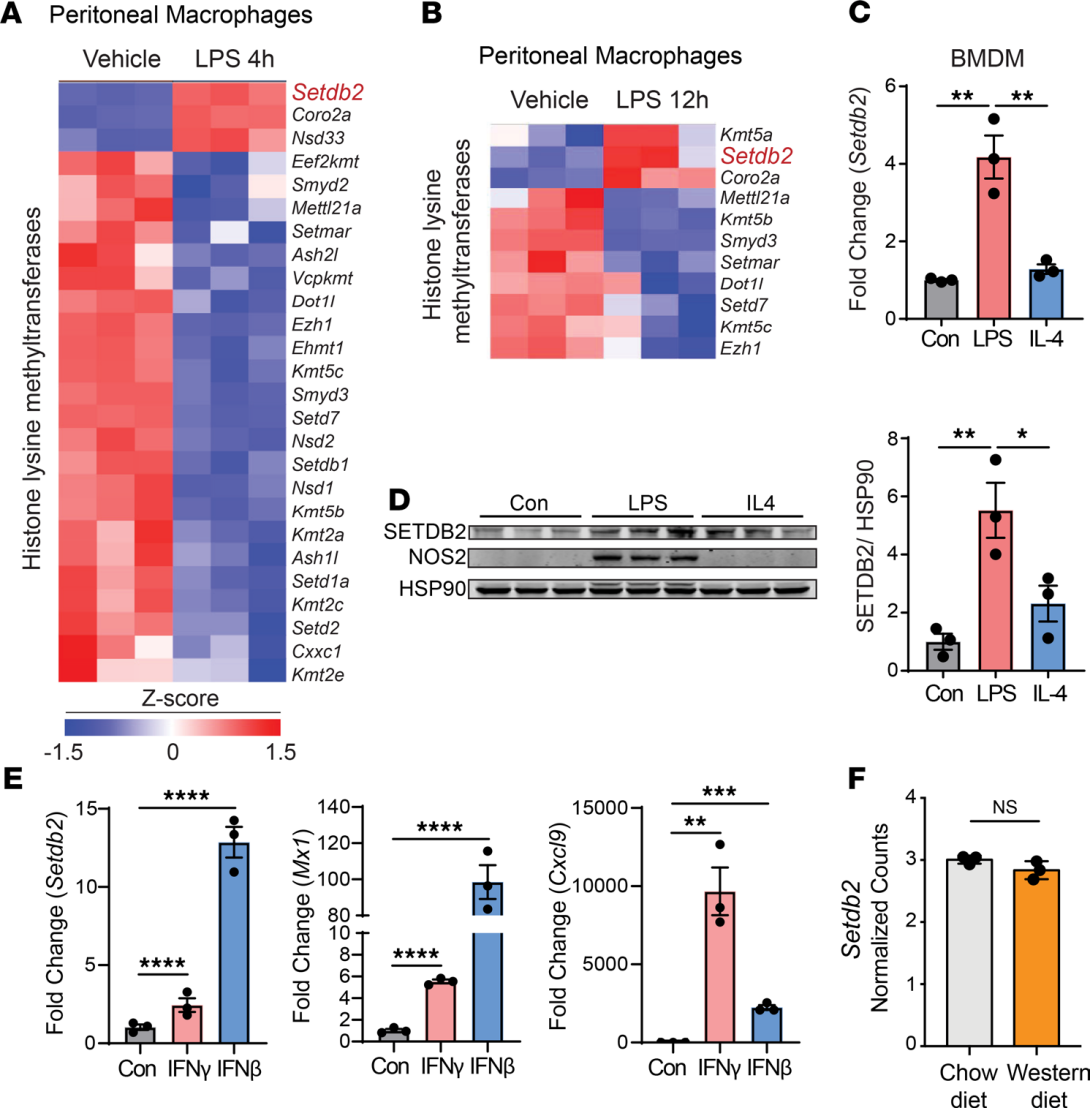
SETDB2 is the most upregulated histone methyltransferase in classically activated macrophages. (**A** and **B**) Heatmaps representing expression (rlog-transformed values) of genes differentially expressed in WT macrophages treated without or with LPS at 4 (**A**) and 12 (**B**) hours. (**C**) qRT-PCR analysis of *Setdb2* expression in BM-derived macrophages (BMDM) from WT mice treated with LPS (classical macrophages) or IL-4 (alternative macrophages) for 12 hours. Data represent the mean ± SEM of relative expression levels normalized to control. **P < 0.01. (**D**) Western blot analysis of SETDB2 in BMDM from WT and *Setdb2*^GT^ mice treated with LPS (classical macrophages) or IL-4 (alternative macrophages) for 24 hours. HSP90 was used as a loading control. Quantification represents the mean ± SEM of relative expression levels normalized to control. *P < 0.05, **P < 0.01. (**E**) qRT-PCR analysis of *Setdb2* expression in BMDM from WT mice treated with IFN-γ (5 ng/ml) and IFN-β (10 ng/ml) for 12 hours. Data represent the mean ± SEM of relative expression levels normalized to control. **P < 0.01, ***P < 0.001, ****P < 0.0001. (**F**) RNA-Seq analysis of *Setdb2* expression in mouse peritoneal macrophages from *Ldlr^–/–^* mice fed a chow diet or Western diet for 12 weeks. Data are shown in representative counts from 3 independent experiments. Data were analyzed by 1-way ANOVA and Tukey’s post hoc test (**C**–**E**) or unpaired 2-tailed Student’s *t* test (**F**).

Activation of the innate immune response in the arterial wall plays a major role in the pathogenesis of atherosclerosis. Macrophage activation in response to pathogen-associated molecular patterns and damage-associated molecular patterns is responsible for initiating and perpetuating the chronic inflammatory cascade that characterizes this disease. To determine how innate immune activation influences the expression of histone-modifying enzymes, we treated mouse peritoneal macrophages with LPS for 4 and 12 hours. Notably, SETDB2 was the most upregulated histone-modifying enzyme in response to LPS at both time points (Figure 1, A and B). Similarly, SETDB2 was also upregulated at both the mRNA and protein levels in BM-derived macrophages (BMDMs) treated with LPS, but SETDB2 expression was refractory to IL-4 treatment, indicating that it is not part of the alternative macrophage activation pathway (Figure 1, C and D, and Supplemental Table 1; supplemental material available online with this article; https://doi.org/10.1172/jci.insight.147984DS1). LPS-mediated TLR4 signaling elicits two parallel signaling pathways: the MyD88 pathway, which triggers NF-κB and AP-1 activation, TLR-stimulated genes, and related inflammatory cytokine production, and the TIR domain–containing adapter-inducing IFN-β pathway, which activates IRF3/7, which mediates the subsequent upregulation of genes encoding type I IFNs and costimulatory molecules. SETDB2 is known to be an IFN-response gene, which can be regulated by both type I and type II IFN. (8, 9, 11) The upregulation of *Setdb2* by IFN-γ and IFN-β in peritoneal macrophages was confirmed by qRT-PCR analysis (Figure 1E). Interestingly, IFN-β–induced *Setdb2* expression was significantly higher compared with IFN-γ expression (Figure 1E), suggesting that LPS-induced SETDB2 expression in macrophages is likely mediated by type I IFN signaling.

In addition to innate immune activation, lesional macrophages engulf large amounts of modified lipoproteins, which leads to the accumulation of foamy cells in atherosclerotic plaques. Thus, we studied whether lipid loading might influence macrophage expression of SETDB2. To this end, we assessed the expression of histone-modifying enzymes in macrophages in vivo under hyperlipidemic conditions induced by high-fat diet and genetic absence of the low-density lipoprotein receptor (*Ldlr^–/–^*). This approach allows the analysis of changes in lipid metabolism and gene expression during foam cell formation in vivo. RNA-Seq analysis revealed no significant changes in the expression of histone-modifying enzymes, including *Setdb2*, in foamy macrophages compared with macrophages isolated from normolipidemic mice (Figure 1F). Together, these findings demonstrate that SETDB2 expression is upregulated upon innate immune activation but is not affected by lipid loading.

### SETDB2 is highly expressed in mouse and human atherosclerotic plaques.

**Figure 2:**
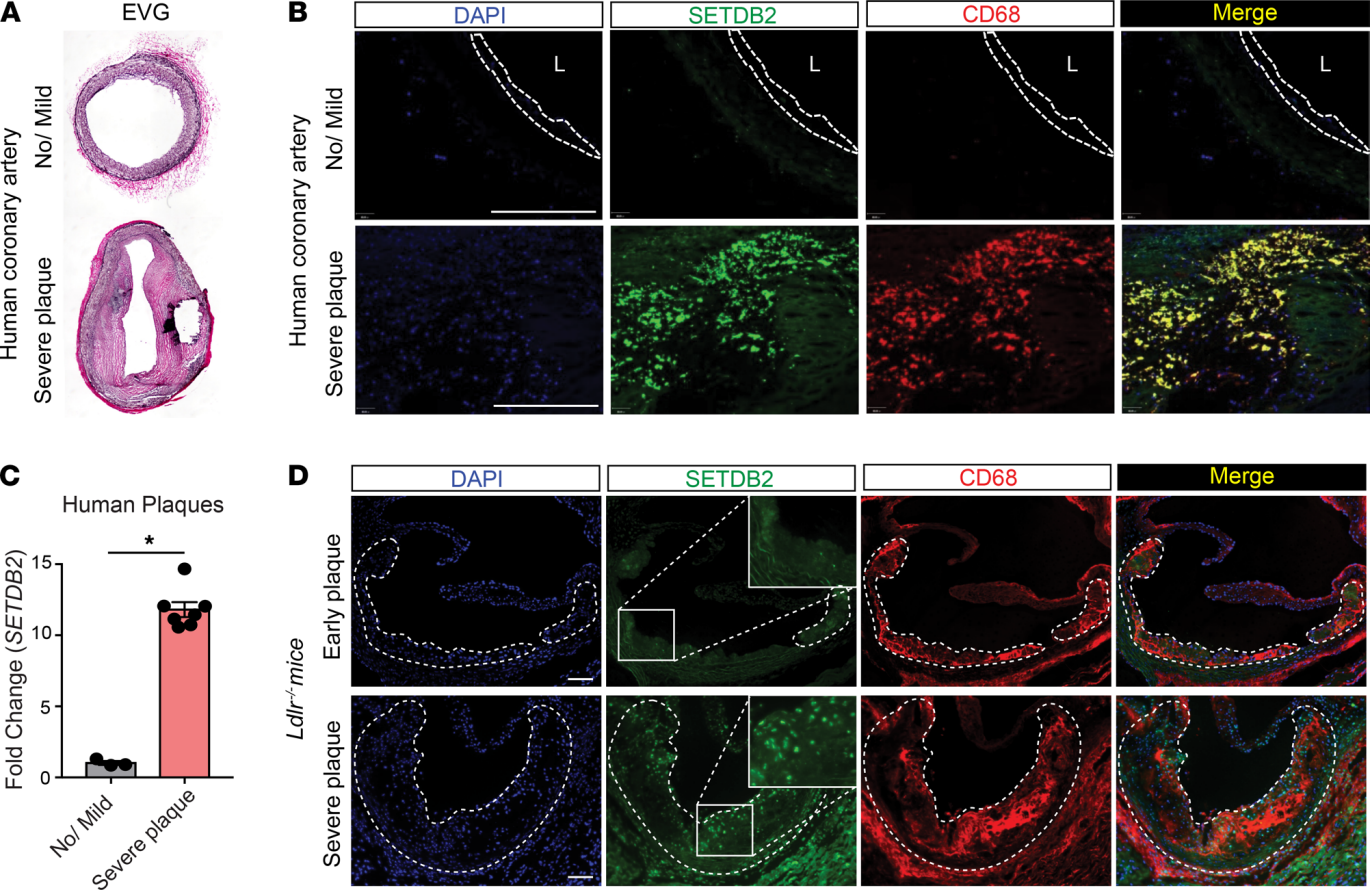
SETDB2 is highly expressed in atherosclerotic macrophages. (**A**) Representative elastic van Gieson (EvG) staining from healthy (top) and atherosclerotic (bottom) human coronary arteries. (**B**) Representative immunofluorescence analysis of SETDB2 and CD68 expression in human coronary lesions. (**C**) qRT-PCR analysis of *Setdb2* expression in healthy (no/mild, *n =* 3) and atherosclerotic arteries (severe plaque, *n =* 7). Quantification represents the mean ± SEM of relative expression levels normalized to healthy artery. *P < 0.05. Data were analyzed by an unpaired 2-tailed Student’s *t* test. (**D**) Representative immunofluorescence analysis of SETDB2 and CD68 expression in mouse atherosclerotic plaques. Dashed bars indicate the atherosclerotic plaques. Scale bar: 100 μm. L, lumen.

We next analyzed the expression of SETDB2 in atherosclerotic human coronary arteries and mouse aortic sinuses (Figure 2). Notably, we found the highest expression of SETDB2 in intimal macrophages from human atherosclerotic plaques (Figure 2, A and B). This finding correlates with the higher mRNA expression level of *SETDB2* in lysates from atherosclerotic lesions compared with healthy human coronary arteries (Figure 2C). Similar to the results observed in human plaques, SETDB2 expression was also highly abundant in mouse lesional macrophages (Figure 2D).

### Hematopoietic SETDB2 deficiency promotes the progression of atherosclerosis.

**Figure 3:**
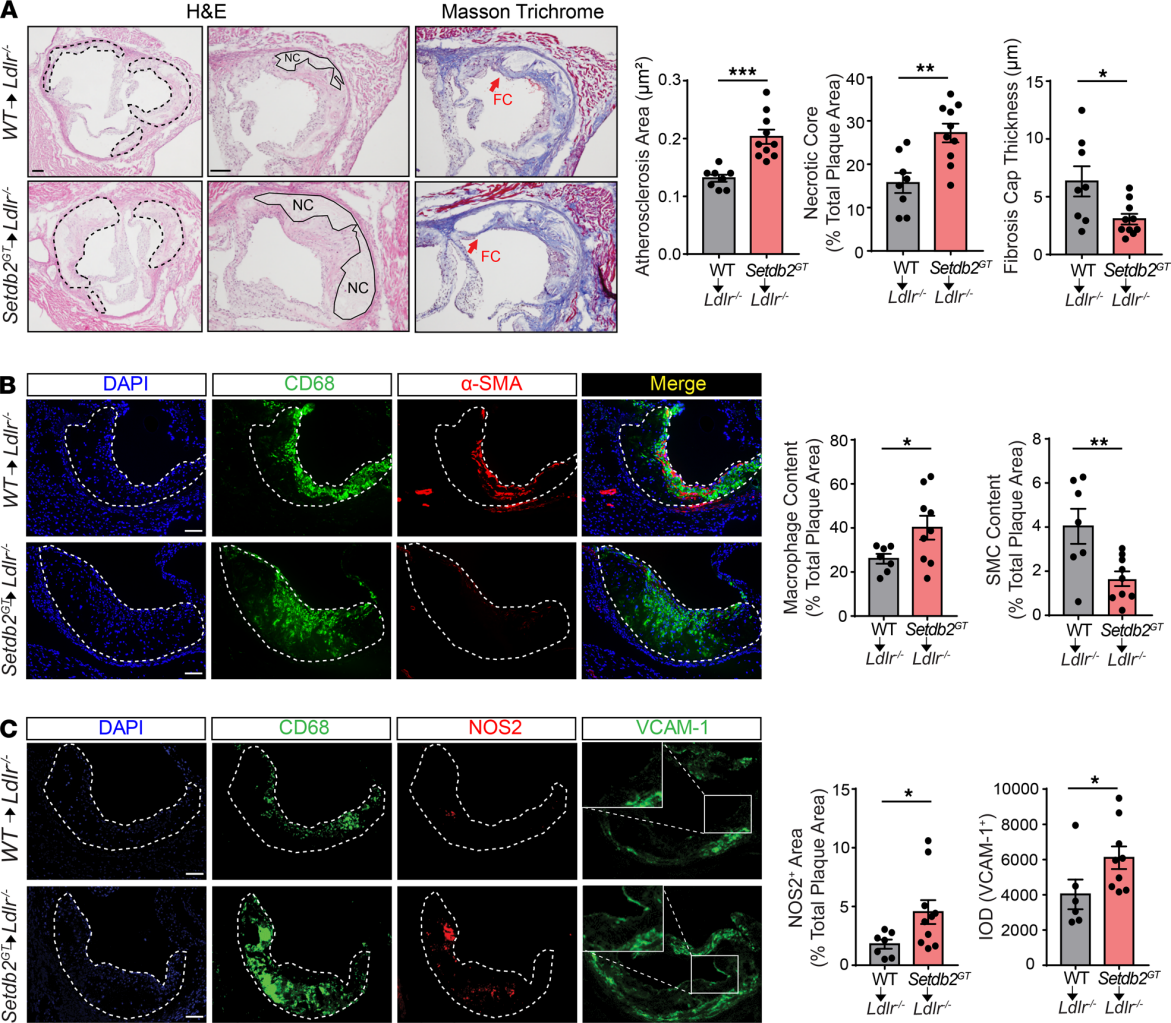
Loss of SETDB2 in hematopoietic cells promotes atherosclerosis progression and plaque instability. (**A**) Representative histological analysis of cross-sections of the aortic sinus stained with H&E (left) and Masson’s trichrome (right) in *Ldlr^–/–^* mice transplanted with WT or *Setdb2*^GT^ BM fed a Western diet for 14 weeks. Means of the lesion area, necrotic core (NC) area, and fibrous cap (FC) thickness calculated from H&E-stained and Masson’s trichrome–stained aortic cross-sections were quantified and are shown (*n =* 8–10 mice per group). Dashed lines indicate the atherosclerotic lesion, and continuous lines indicate the boundary of the developing NC (plaque acellular area). (**B** and **C**) Representative immunofluorescence analysis of aortic root cross-sections of the aortic root lesions stained with CD68 (macrophage marker) and smooth muscle α-actin (α-SMA) (**B**) as well as NOS2 and VCAM-1 (**C**) in *Ldlr^–/–^* mice transplanted with WT or *Setdb2*^GT^ BM fed a Western diet for 14 weeks. Dashed lines indicate the atherosclerotic lesion. Quantifications are shown (*n =* 8–10 mice per group). All data represent mean ± SEM. *P < 0.05, **P < 0.01, ***P < 0.001 compared with *Ldlr^–/–^* mice transplanted with WT BM. Data were analyzed by an unpaired 2-tailed Student’s *t* test. Scale bar: 100 μm.

To determine the in vivo function of SETDB2 during the progression of atherosclerosis, we developed mice with a gene trap insertion at the *Setdb2* locus that is designed to limit global expression of the *Setdb2* gene (*Setdb2*^GT^ mice). (10) We transplanted BM cells from WT or *Setdb2*^GT^ mice to lethally irradiated male *Ldlr^–/–^* mice followed by a Western diet (WD, 40% fat and 1.25% cholesterol) for 14 weeks. While mice transplanted with *Setdb2*^GT^ BM showed a statistically significant increase in body weight (Supplemental Figure 1A), plasma cholesterol, high-density lipoprotein cholesterol, triglycerides, blood glucose, and plasma leukocytosis were similar in both groups of mice (Supplemental Figure 1, B–F). Compared with *Ldlr^–/–^* mice transplanted with WT BM, after 14 weeks on a WD, *Setdb2* deficiency in hematopoietic cells resulted in larger atherosclerotic lesions analyzed in the aortic root (Figure 3A, quantified on the right). Notably, we observed a significantly larger necrotic core (NC) size and thinner fibrous cap, two morphological markers of vulnerable atherosclerotic lesions, in mice receiving BM from *Setdb2*^GT^ donors compared with mice transplanted with WT BM (Figure 3A, quantified on the right). Detailed histological analysis of the aortic root plaques revealed a significant increase in macrophage content (CD68^+^ cells) in the lesions (Figure 3B) accompanied by enhanced vascular inflammation analyzed by immunostaining of inducible nitric oxide synthase (iNOS) and vascular cell adhesion molecule-1 (VCAM-1) in mice lacking SETDB2 in hematopoietic cells (Figure 3C). As expected by the greater NC and thinner fibrous cap found in mice transplanted with *Setdb2*^GT^ BM, we found a significant reduction of α-SMA^+^ vascular smooth muscle cell coverage in lesions from *Ldlr^–/–^* mice receiving *Setdb2*^GT^ BM (Figure 3B). No differences were found in lipid deposition in atherosclerotic lesions (Supplemental Figure 2A); this finding correlates with the similar foam cell formation observed in peritoneal macrophages isolated from *Ldlr^–/–^* mice transplanted with WT and *Setdb2*^GT^ BM and fed a WD for 12 weeks (Supplemental Figure 2B) and BMDMs treated with acetylated LDL (Supplemental Figure 2C). Together, these findings demonstrate that loss of *Setdb2* in hematopoietic cells accelerates the progression of atherosclerosis and promotes plaque instability by increasing inflammation and macrophage accumulation in the lesions.

### Single-cell transcriptomics analysis reveals an increased accumulation of proinflammatory macrophages in atherosclerotic lesions from mice lacking SETDB2 in hematopoietic cells.

**Figure 4:**
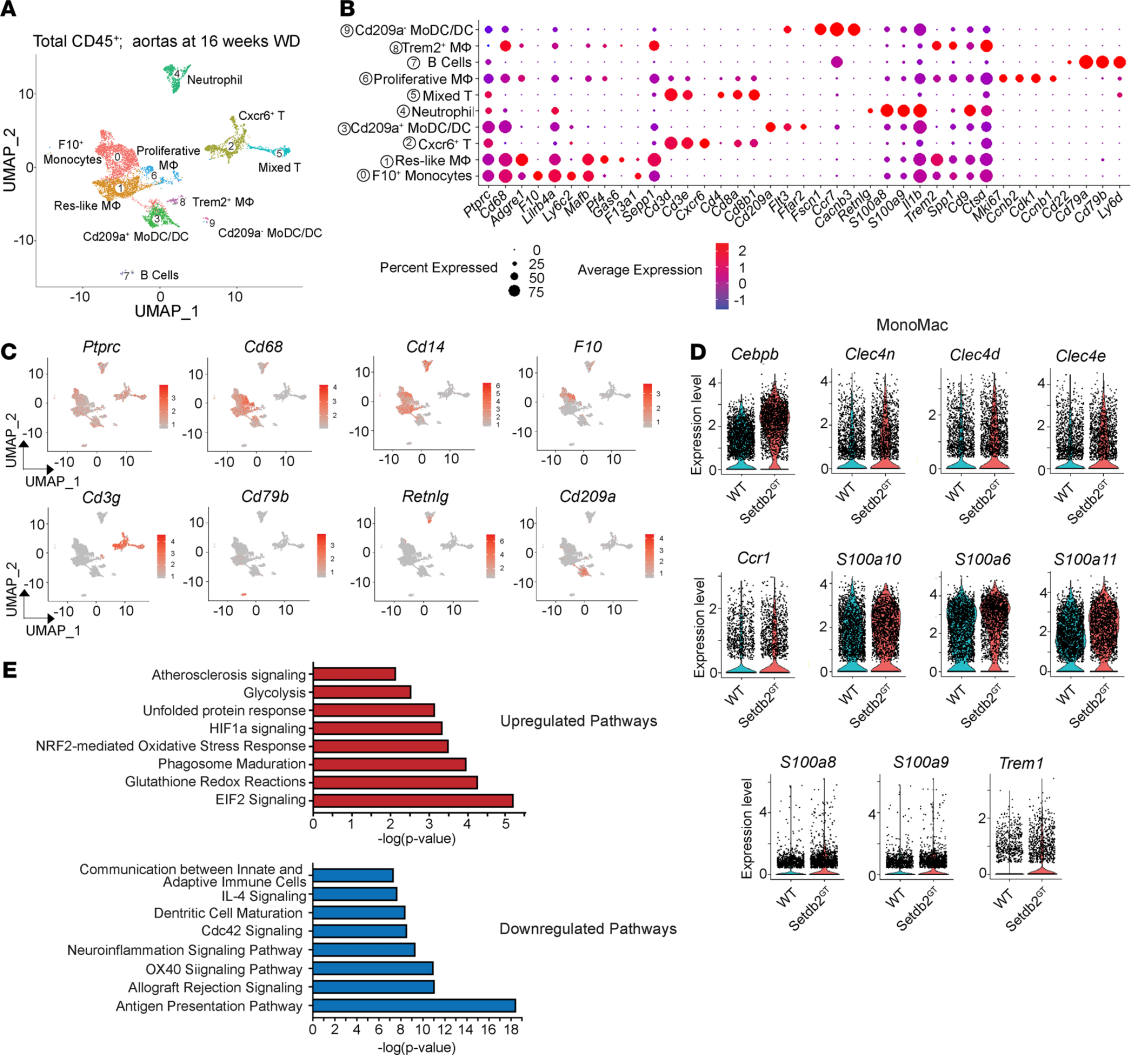
Single-cell RNA-Seq analysis reveals increased monocyte recruitment and vascular inflammation in atherosclerotic plaques isolated from *Ldlr^–/–^* mice transplanted with *Setdb2^GT^* BM cells. (**A**) Uniform manifold approximation and projection (UMAP) representation of aligned gene expression data in single cells extracted from atherosclerotic aortas of *Ldlr^–/–^* mice transplanted with WT (5149 cells) or *Setdb2*^GT^ (4159 cells) BM after 16 weeks Western diet feeding. UMAP analysis identified 10 major clusters. (**B**) Feature plots depicting single-cell gene expression of individual genes. (**C**) Dot plots depicting diverse gene expression with known markers of the monocytes, macrophages, T cells, dendritic cells, neutrophils and B cells. (**D**) Violin plots of the top differentially expressed transcripts, showing statistically marked upregulation in the *Setdb2*^GT^ monocyte/macrophage population. (**E**) Pathway enrichment of differentially expressed transcripts defined in **D** expressed as the log[–P] analyzed by Ingenuity Pathway Analysis.

To investigate the molecular and cellular basis of how SETDB2 expression in hematopoietic cells influences the progression of atherogenesis, we performed scRNA-Seq of CD45^+^ cells isolated from atherosclerotic plaques. To this end, CD45.2 BM cells isolated from WT and *Setdb2*^GT^ mice were transplanted to lethally irradiated CD45.1 *Ldlr^–/–^* mice. Atherosclerotic plaques were digested into single cells after 14 weeks of WD feeding, and CD45.2^+^ leukocytes were isolated by fluorescence-activated cell sorting. A total of 5149 WT and 4159 *Setdb2*-deficient cells were sequenced, and the analysis identified 10 distinct cell clusters based on gene expression patterns of established canonical markers of lymphocyte lineages (*Cd3g* and *Cd79b*) and myeloid cells (*Cd14*, *Cd68*, *Lgals3*, *Ccr2*, *Ly6c2*, *Retnlg*, and *Cd209a*; Figure 4, A–C; Supplemental Figure 3; and ref. 12). Consistent with increased macrophage content observed in lesions from *Setdb2*^GT^ BM–transplanted mice observed by immunostaining in Figure 3, scRNA-Seq analysis revealed increased F10^+^ monocytes (cluster 0) in the atherosclerotic lesions from *Ldlr^–/–^* mice transplanted with *Setdb2*^GT^ BM (Supplemental Figure 4A). A significant number of transcripts from genes involved in the proinflammatory response (*Cebpb*, *Tyrobp*, *Trem1*, *Il1r2*, *Il1b*, *Il1a*, and *Tnf*) and unfolded protein response (*Clec4e* and *Clec4d*) were upregulated in *Setdb2*-deficient leukocytes (Supplemental Figure 4B and Supplemental Tables 2 and 3), which is consistent with the in vivo phenotype of increased inflammation and plaque instability in *Setdb2*-deficient atherosclerotic lesions.

SETDB2 has been identified as a novel regulator of a subset of proinflammatory genes involved in monocyte and neutrophil recruitment by increasing H3K9 trimethylation levels at promoters of *Ccl2* and *Cxcl1* (8, 9) during the resolution phase of the proinflammatory response. In our study, we found increased monocytes (F10^+^, cluster 0) and neutrophil (cluster 4) in *Setdb2*-deficient leukocytes compared with WT cells (Supplemental Figure 4A). This was accompanied by increased expression of proinflammatory molecules (*Cebpb*, *S100a8*, *S100a9*, *Ccr1*, and *Trem1*) and unfolded protein response (*Clec4e*, *Clec4d*, and *Clec4n*) in the monocyte/macrophage population (clusters 0, 1, and 6) from *Setdb2*^GT^ BM–transplanted mice (Figure 4D and Supplemental Tables 4–9). Pathway enrichment analysis of these differentially upregulated transcripts was associated with biological processes involved in protein synthesis (EIF2 signaling), oxidative stress (glutathione redox reactions and NRF2-mediated oxidative stress response), cell apoptosis (unfolded protein response), and atherosclerosis signaling (Figure 4E). Furthermore, pathway analysis of differentially downregulated transcripts demonstrated impaired regulation of antigen presentation pathway and antiinflammatory response (IL-4 signaling; Figure 4E) in the monocyte/macrophage population.

### Setdb2 deficiency in macrophages enhances inflammatory response and impairs efferocytosis.

**Figure 5:**
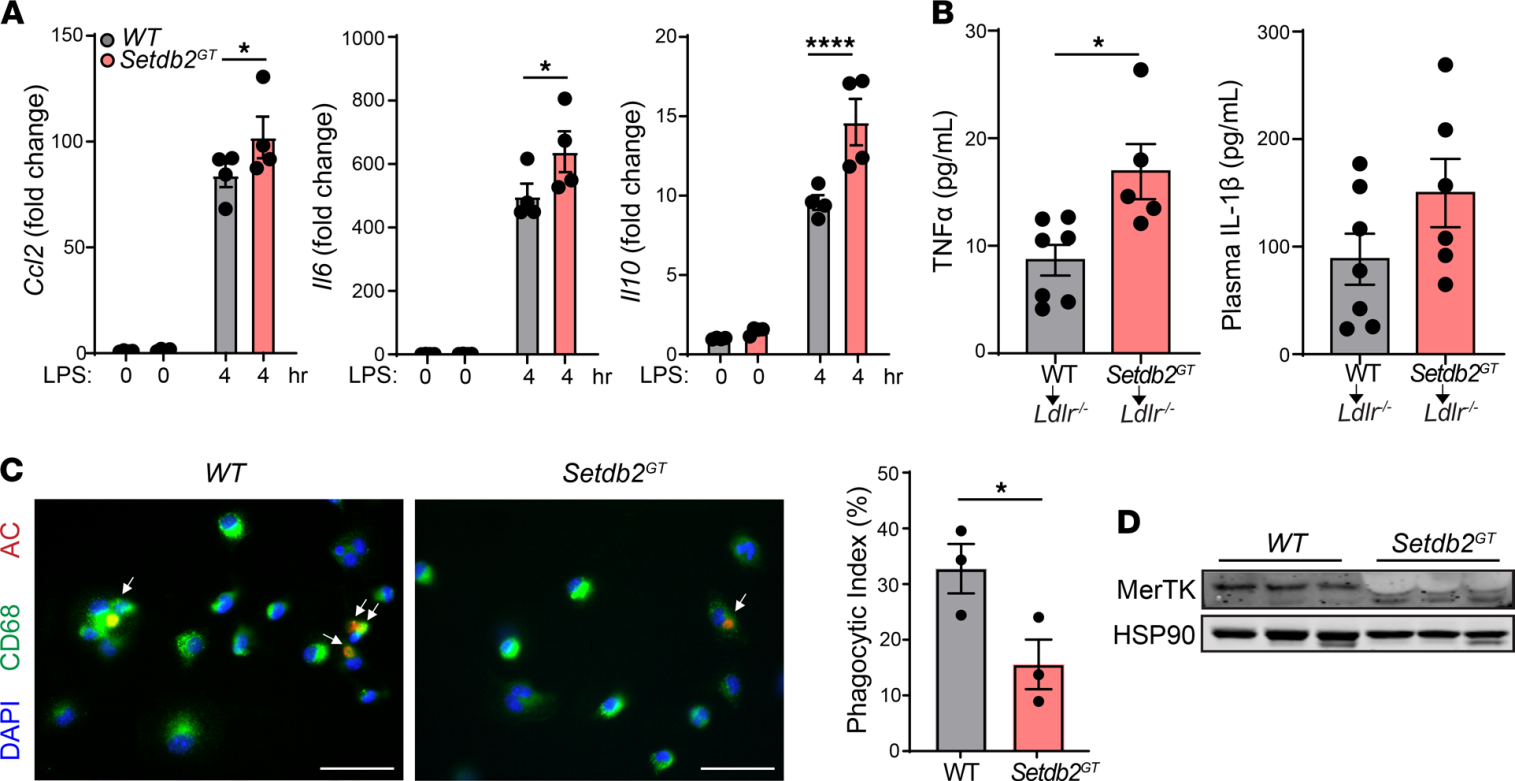
Macrophage *Setdb2* deficiency enhances inflammatory response and impairs macrophage efferocytosis. (**A**) qRT-PCR analysis of LPS-induced (100 ng/ml for 4 hours) expression of *Ccl2*, *IL-6*, and *IL-10* in BMDMs isolated from WT or *Setdb2*^GT^ mice (*n =* 4 per group). Data were analyzed by 1-way ANOVA and Tukey’s post hoc test. (**B**) Analysis of plasma *Il1b* and *Tnfa* levels from *Ldlr^–/–^* mice transplanted with WT or *Setdb2*^GT^ BM and fed a Western diet for 14 weeks (*n =* 5–7). Data were analyzed by an unpaired 2-tailed Student’s *t* test. (**C**) Representative images (left) of the in vitro engulfment of CellTracker Red–labeled apoptotic Jurkat cells (AC) by BM-derived macrophages (BMDMs) isolated from WT or *Setdb2*^GT^ mice. Quantification is shown (*n =* 3 each group). Phagocytosis is expressed with the phagocytic index, which is the number of apoptotic cells (red) ingested in 1 hour per CD68^+^ macrophage (green) × 100 of apoptotic Jurkat T cells. Data were analyzed by an unpaired 2-tailed Student’s *t* test (*n =* 3 each group). (**D**) Western blot analysis of MerTK in BMDMs from WT and *Setdb2*^GT^ mice. HSP90 was used as a loading control. *P < 0.05, ****P < 0.0001 compared with WT BMDM or *Ldlr^–/–^* mice transplanted with WT BM.

To determine whether SETDB2 deficiency influences macrophage inflammatory response in vitro, we treated WT and *Setdb2*^GT^ BMDMs with LPS and assessed the expression of *Ccl2*, *Il6*, and *Il10*. The results showed that loss of SETDB2 resulted in a significant increase of *Ccl2*, *Il6*, and *Il10* expression (Figure 5A). In addition to the increased self-intrinsic inflammatory response of *Setdb2*-deficient macrophages and the enhanced plaque inflammation observed in *Ldlr^–/–^* mice transplanted with *Setdb2*^GT^ BM, we also observed a marked increase in circulating proinflammatory cytokines, including TNF-α and IL-1β (Figure 5B). Together, these findings demonstrate that SETDB2 expression in macrophages is associated with the regulation of proinflammatory responses during macrophage activation and systemic inflammation.

Morphological plaque analysis also showed a marked increase in necrotic cores in lesions of mice receiving BM from *Setdb2*-deficient mice (Figure 3A). Larger necrotic cores in atherosclerotic lesions are associated with an increased rate of macrophage apoptosis and impaired efferocytosis. While we did not find differences in apoptotic macrophages (TUNEL^+^CD68^+^ cells) in atherosclerotic plaques from *Ldlr^–/–^* mice transplanted with *Setdb2*-deficient BM (Supplemental Figure 5A), we did find significantly impaired in vitro phagocytotic capacity associated to reduced levels of c-Mer tyrosine kinase (MerTK), a receptor that mediates apoptotic cell engulfment in atherosclerotic lesions (Figure 5, C and D). Moreover, there was also a trend toward lower MerTK expression in lesional macrophages (CD68^+^ cells) from mice transplanted with *Setdb2*-deficient BM (Supplemental Figure 5B). Together, these results suggest that the increased macrophage content in atherosclerotic plaques in mice lacking SETDB2 in hematopoietic cells is likely due to increased macrophage recruitment and decreased clearance, as the number of proliferative macrophages was reduced in lesions isolated from these animals (Supplemental Figure 6).

## Discussion

Overall, the major finding of this study is the identification of SETDB2 as a major regulator of immune activation during atherosclerosis. Absence of *Setdb2* in hematopoietic cells promotes vascular inflammation and enhances atherosclerotic lesion burden. Mechanistically, this phenotype is due to an increase in proatherogenic pathways, such as enhanced innate immune activation, impairment in resolution of inflammation, and increased vascular inflammation. We also report that SETDB2 is the most upregulated methyl transferase enzyme in response to LPS and, consistent with previous studies, show that SETDB2 is regulated by IRF7 downstream of IFN (9). These findings correlate with recent studies showing the antiinflammatory effect of SETDB2 in both a model of diabetic wound healing and during an acute respiratory viral infection (9, 11). Our histological analysis and scRNA-Seq data demonstrate that SETDB2 is highly expressed in lesional macrophages and its absence enhances the expression of a number of proinflammatory cytokines, including *Nos2*, *Il1b*, *Tnfa*, and *Cxcl2*. The increased production of *Ccl2*, *Il6*, and *Il10* in *Setdb2*-deficient macrophages is likely mediated by the reduction of H3K9m3 marks in the promoter of these genes, which is associated with transcriptional inhibition (13, 14). Indeed, Gallagher and colleagues have recently demonstrated that absence of SETDB2 in macrophages significantly reduced levels of H3K9m3 at the NF-κB–binding site of the *Il1b*, *Tnfa*, and *Nos2* in *Setdb2*-deficient macrophages compared with littermate controls (11). In addition to the regulation of *Il1b* expression, absence of SETDB2 enhanced expression of the enzyme xanthine dehydrogenase (XOR), which would reduce xanthine levels and increase uric acid production (11). Uric acid activates the nod-like receptor protein-3 (NLRP3) inflammasome, resulting in increased production of IL-1β. However, we were unable to find differences of inflammasome activation in vitro (data not shown).

In this study, we also demonstrate that reduced STEDB2 in hematopoietic cells promotes a significant accumulation of macrophages in atherosclerotic plaques that is associated with increased monocyte recruitment and impaired efferocytosis. Notably, SETDB2 has been reported to be involved in the regulation of cell cycle progression in acute lymphoblastic leukemia but does not have any effect on normal hematopoietic stem and progenitor cell proliferation (15). This finding correlates with the decreased macrophage proliferation found in atherosclerotic lesions from *Ldlr^–/–^* mice transplanted with *Setdb2*-deficient versus WT BM and similar levels of circulating leukocytes in our study. In addition to changes in macrophage proliferation, we also found greater necrosis in plaques from *Ldlr^–/–^* mice transplanted with *Setdb2*^GT^ BM compared with *Ldlr^–/–^* mice reconstituted with WT BM, which is associated with increased macrophage apoptosis and impairment in efferocytosis (16, 17). Notably, we found a significant reduction in efferocytotic capacity in macrophages lacking SETDB2 compared with WT macrophages; this finding correlates with a significant reduction of MerTK, a key receptor that controls apoptotic cell engulfment in atherosclerotic lesions (18). The mechanism behind this finding requires further investigation but may be explained by SETDB2-mediated epigenetic regulation of macrophage fate from an inflammatory to a reparative phenotype.

In addition to its role in regulating epigenetic silencing of gene expression, SETDB2 can also positively regulate gene expression, acting as a scaffolding protein that facilitates the binding of transcription factors to enhancers and promoter regions (10). In this regard, we have shown that SETDB2 promotes GR binding to *Insig2a* enhancers, leading to an increase in expression of INSIG2a in liver during fasting (10). This results in a retention of the SCAP/SREBP complex in the ER and suppression of lipogenesis under fasting conditions (10). While these observations identified a possibly novel role for SETDB2 in regulating lipid metabolism, we did not observe differences in neutral lipid accumulation in aortic lesions and macrophage foam cell formation in mice. Further studies will be needed to assess the nonenzymatic function of SETDB2 in regulating macrophage functions during atherosclerosis.

One caveat of our study is the use of BM transplantation studies to assess the role of the immune system in atherosclerosis (19, 20). This approach does not dissect the specific contribution of SETDB2 in macrophages during atherogenesis. SETDB2 is also expressed in T cells that might play a role in the adaptive immune response in the lesions. Indeed, our scRNA-Seq analysis showed a significant reduction in T cell populations in atherosclerotic aortas isolated from *Ldlr^–/–^* mice transplanted with *Setdb2*^GT^ BM. This can also be explained by the function of SETDB2 in macrophages, which has been shown to regulate T cell proliferation in virally induced lung damage (9).

In summary, we identified SETDB2 as an important regulator of macrophage function and inflammatory response during atherosclerosis. Our results also suggest that enhanced activation of SETDB2 in atherosclerotic lesions will attenuate chronic inflammation and facilitate tissue repair and inflammation resolution, offering a therapeutic approach to treat atherosclerotic vascular disease.

## Methods

Detailed methods are provided in the Supplemental Methods.

### RNA-Seq and scRNA-Seq data

Data were deposited in the NCBI’s Gene Expression Omnibus database (GEO GSE169112 and GSE168777).

### Statistics

All data are expressed as mean ± SEM. Statistical differences were calculated with unpaired 2-tailed Student’s *t* test, 1-way ANOVA (followed by the Newman-Keuls post test), or 2-way ANOVA (followed by the Bonferroni post test). A *P* value of less than 0.05 was considered statistically significant for all analyses. Data analysis was performed using GraphPad Prism software version 7.0.

### Study approval

#### Animal experiments.

BM cells were obtained by flushing the tibias and femurs of age-matched (8 weeks old) WT or *Setdb2*^GT^ mice on C57BL/6J backgrounds, as described previously (21). The transplantation was done in *Ldlr^–/–^* mice lethally irradiated with 5 Gy 2 times in series and intravenously infused with 2 × 10^6^ donor BM cells for each mouse (22, 23). At 4 weeks after the transplantation, by which time the BM of the recipient mice was reconstituted, atherosclerosis was induced by feeding the mice a WD (40% fat and 1.25% cholesterol, no. D12108, Research Diets Inc.) for 14 weeks (24, 25). Mice used in all experiments were sex and age matched and kept in individually ventilated cages under pathogen-free conditions. All of the experiments were approved by the Institutional Animal Care Use Committee of Yale University School of Medicine.

#### Human subjects.

Human left main coronary arteries were obtained from the explanted hearts of transplant recipients or cadaver organ donors (Supplemental Table 10 and ref. 26). Research protocols were approved by the institutional review boards of Yale University and the New England Organ Bank (Waltham, Massachusetts, USA). A waiver for consent for surgical patients was approved by Yale’s Human Investigation Committee, and written informed consent was obtained from a member of the family for deceased organ donors by the New England Organ Bank.

## Author contributions

CFH, TFO, YS, and XZ conceived and designed the study. XZ, JS, ACD, BA, and YJC performed experiments and analyzed data. GT collected human specimens. YS, TFO, and CFH assisted with experimental design and data interpretation. CFH and XZ wrote the manuscript, which was reviewed by all authors.

## Supplementary Material

Supplemental data

## Version 1 05/18/2021

In-Press Preview

## Version 2 06/22/2021

Electronic publication
