# Supplementary material for: Deficiency of histone lysine methyltransferase SETDB2 in hematopoietic cells promotes vascular inflammation and accelerates atherosclerosis
Source: JCI Insight. 2021 Jun 22;6(12):e147984. doi: 10.1172/jci.insight.147984 (PMC8262461; doi:10.1172/jci.insight.147984)
Supplement: Supplemental data [file jciinsight-6-147984-s157.pdf]

Deficiency of histone lysine methyltransferase SETDB2 in hematopoietic cells promotes vascular inflammation and accelerates atherosclerosis

Xinbo Zhang[1,2], Jonathan Sun[1,2], Alberto Canfrán-Duque[1,2], Binod Aryal[1,2,], George Tellides[4], Ying Ju Chang[,4,5], Yajaira Suárez[1,2,#], Timothy F. Osborne[5,6,#], Carlos Fernández-Hernando[1,2#.]

METHODS

Bone marrow transplantation experiments. Bone Marrow (BM) cells were obtained by flushing the tibias and femurs of age-matched (8-week-old) wild type (WT) or *Setdb2*^{GT} mice as described previously (1). The transplantation was done in *Ldlr*$^{-/-}$ mice lethally irradiated with 5 Gy two times in series and intravenously infused with 2×10^6 donor BM cells each mouse. At 4 weeks after the transplantation, by which time the BM of the recipient mice was reconstituted, atherosclerosis was induced by feeding the mice a Western-type diet (WD, 40% fat and 1.25% cholesterol, #D12108, Research Diets, Inc., New Brunswick, NJ) for 14 weeks. Mice used in all experiments were sex and age matched, kept in individually ventilated cages in pathogen-free. All of the experiments were approved by the Institutional Animal Care Use Committee of Yale University School of Medicine.

Plasma lipids analysis

Before and after WD feeding, mice were fasted for 12-14 h before blood samples were collected by retro-orbital venous plexus puncture. Plasma was separated by centrifugation and stored at -80°C until analysis. Plasma total cholesterol (TC), high density lipoprotein-cholesterol (HDL-C) and triglyceride (TG) concentrations were determined by standard enzymatic methods (Wako Chemicals, USA).

Histology and morphometric analysis

Following anesthesia (100 mg/kg ketamine; 10 mg/kg xylazine), thoracic cavity was exposed immediately and in situ perfusion fixation through the left cardiac ventricle was performed by thorough perfusion with

PBS and 4% paraformaldehyde (PFA). Subsequently, hearts and aortas were harvested and fixed in 10% formaldehyde solution overnight. Hearts were embedded in OCT after dehydration with 30% sucrose and serial sections were cut at 6 μm thickness using a cryostat. Every third slide from the serial sections was stained with haematoxylin and eosin (H&E) and each consecutive slide was stained with oil red O (ORO) for quantification of lesion area. Aortic lesion size of each animal was obtained by averaging the lesion areas in four sections from the same mouse. The necrotic core area was measured as a percentage of the total plaque area from the three sections from the same mouse. Collagen content was assessed by Masson's Trichrome staining of consecutive slides from serial sections and quantified as a percentage of the total plaque area.

Immunofluorescence staining

The atherosclerotic sections were fixed with 4% PFA and incubated overnight with primary antibodies for Setdb2 (Abcam, #ab133174), CD68 (Serotec; #MCA1957), NOS2 (Cell Signaling, #2982), VCAM-1 (Abcam; ab19569) and Ki67 (Abcam, #ab15580) after blocking with blocker buffer (5% Donkey Serum, 0.5% BSA, 0.3% Triton X-100 in PBS) for 1 hour at RT, followed by incubation with Alexa Fluor secondary antibody (Invitrogen, Carlsbad, CA) for 1 hour at RT. The stained sections were captured using a Carl Zeiss scanning microscope Axiovert 200M imaging system and images were digitized under constant exposure time, gain, and offset. Results are expressed as the percent of the total plaque area stained measured with the Image J software.

Blood leukocytes analysis

Blood was collected by retro-orbital puncture in heparinized micro-hematocrit capillary tubes. Erythrocytes were lysed with ACK lysis buffer (155 mM Ammonium Chloride, 10 mM Potassium Bicarbonate, 0.01 mM EDTA, pH 7.4). White blood cells (WBC) were resuspended in 3% FBS in PBS, blocked with 10 μg/ml of FcgRII/III (Biolegend), then stained with a cocktail of antibodies. Monocytes were identified as $CD115^{hi}$ and subsets as Ly6-C^{hi} and Ly6-C^{lo}; neutrophils were identified as $CD115^{lo}$Ly6-C^{hi}Ly6-G^{hi}. The following antibodies were used (BioLegend, 1:300 dilution): FITC-Ly6-C (AL-

21), PE-CD115 (AFS98), APC-Ly6-G (1A8). WBC and platelet counting in circulation was determined from EDTA-anticoagulated blood using a hemocytometer (Hemavet Counter HV950FS). For the analysis of leukocytes in aortic tissue, entire aorta was perfused with PBS, cut into small pieces and subjected to enzymatic digestion with 400 U/ml collagenase I, 125 U/ml collagenase XI, 60 U/ml DNase I and 60 U/ml hyaluronidase (Sigma-Aldrich) for 1 h at 37 °C while shacking. Total viable cell numbers were obtained using Trypan Blue (Cellgro).

Aortic CD45$^+$ cell isolation from atherosclerotic lesions

BM cells from WT or *Setdb2*^{GT} (CD45.2) were transplanted to lethally irradiated *Ldlr*$^{-/-}$ (CD45.1) mice and fed a WD diet for 16 weeks to induce atherosclerosis. To obtain the monocytes/macrophages in the atherosclerotic lesions, the aortas were digested (from the root to the diaphragm, n=3 each group) with 1 mg/ml Collagenase A (Roche, Cat 11088785103) for 7 min at 37 °C to remove the adventitia under microscope. Aortic tissue was cut into small pieces and subjected to enzymatic digestion with 1.5 mg/ml Collagenase A and 0.5 mg/ml Elastase (Worthington, Cat LS006365) for 40 min at 37 °C while shaking. The digested aortas were passed through a 70 µm Cell Strainer to obtain single cell suspensions followed by incubation of 10 minutes at 4°C with 10 µg/ml of purified rat anti-mouse FcgRII/III (Biolegend) to block non-specific binding of antibodies to Fc Receptors. Total cell viability was analyzed using live/dead viability dye eFluor 450 (Thermo Fisher Scientific). Cells were subsequently stained with anti-mouse CD45.2-PE (Biolegend, Cat 109808) and anti-mouse CD45.1-FITC (Biolegend, Cat 110706) at 4°C for 30 minutes. Viable CD45.2 cells were sorted by FACS Aria III (BD Biosciences) and immediately processed for single-cell RNA-Seq.

Droplet-based scRNA-seq library construction and sequencing

The sorted CD45$^+$ cells were encapsulated into droplets and processed following manufacturer's specifications using 10X Genomics GemCode Technology. Equal numbers of cells per sample were loaded on a 10x Genomics Chromium controller instrument to generate single-cell Gel Beads in emulsion (GEMs) at Yale Center for Genome Analysis. Lysis and barcoded reverse transcription of polyadenylated

mRNA from single cells were performed inside each GEM followed by cDNA generation using the Single Cell 3' Reagent Kits v2 (10X Genomics). Libraries were sequenced on an Illumina HiSeq 4000 as 2 × 100 paired-end reads.

Single cell RNA-seq data analysis

Sample demultiplexing, aligning reads to the mouse genome (mouse UCSC mm10 reference genome) with STAR and unique molecular identifier (UMI) processing were processed using CellRanger software (version 2.1.1) as previously described.(3) Low quality cells, doublets and potentially dead cells were filtered based on the percentage of mitochondrial genes and number of genes and UMIs expressed in each cell. After filtering we identified 5,149 WT cells (a mean of 21,334 reads per cell, a median of 5,666 UMIs per cell, and a median 1,758 genes per cell) and 4,159 *Setdb2*^{GT} cells (a mean of 25,494 reads per cell, a median of 6,907 UMIs per cell, and a median 1,911 genes per cell) for downstream analysis. Data clustering was performed using Seurat R package (Version 3.0) with filtered genes by barcode expression matrices as inputs.(4) Highly variable genes (HVGs) were calculated using Seurat function *FindVariableGenes* and used for downstream clustering analysis. Principal component analysis (PCA) was performed with *RunPCA* function (Seurat) using HVGs for dimensionality reduction and the number of significant principle components was calculated using *JackStraw* function. We applied the *RunTSNE* function to significant principal components (PCs) identified by *JackStraw* analysis and presented data in two-dimensional coordinates through t-distributed stochastic neighbor embedding (t-SNE) generated by R package ggplot2. Clustering was done through *FindClusters* function using 30 significant PCs with a resolution of 0.3. Significantly differentially expressed genes in a cluster were analyzed using Seurat function *FindAllMarkers,* which were expressed in more than 25% of cells with at least 0.25-fold difference and reach statistical significance of an adjusted p<0.05 as determined by the Wilcox test. Ingenuity Pathway Analysis (Ingenuity Systems QIAGEN, Content version: 47547484, 2019, Redwood City, CA, USA) was used to carry out analyses for pathway with differentially-expressed genes across samples. The significance of canonical pathways was determined by IPA's default threshold [–log. (p-value)>1.3]. Data are deposited in NCBI Gene Expression Omnibus (GSE169112).

RNA sequencing

RNA sequencing was performed in thioglycolate-elicited peritoneal macrophage from *Ldlr*$^{-/-}$ mice and treated or not with LPS for 4 and 12 hours. RNA sequencing was carried out at the Yale Center for Genome Analysis using an Illumina HiSeq 2000 platform (1 x 75bp read length). Trimmed reads were aligned using TopHat2, and transcript abundances and differences calculated using cuffdiff. Gene network and pathway analysis were carried out using Gene Set Enrichment Analysis software (Broad Institute) and Qlucore Omics Explorer v 3.2 (Qlucore AB). Data are deposited in NCBI Gene Expression Omnibus (GSE168777).

En face Oil-red O staining

35 ml Oil-Red O stock solution (0.2% weight/volume in methanol) was mixed with 10 ml of 1 M NaOH and filtered. Aortas opened up longitudinally were briefly rinsed with 78% methanol, stained with 0.16% Oil-Red O solution for 50 min and then destained in 78% methanol for 5 min.(5) The stained sections were captured with Olympus SZX16 stereo microscope and quantified with the Image J program. The lesion area was quantified as percent of Oil-Red O staining area in total aorta area. The lipid content in atherosclerotic plaques of aortic valve were stained with Oil-Red O and quantified as a percentage of the total plaque area.

Terminal Deoxynucleotidyl Transferase End Labeling (TUNEL)

Apoptotic cells in lesions were labeled by TUNEL using the *in situ* cell death detection kit TMR-red (Roche Diagnostics) according to the manufacturer's protocol. Briefly, frozen sections were fixed with 10% PFA and incubated with permeabilisation solution (0.1% Triton X-100, 0.1% sodium citrate in PBS buffer) for 2 min on ice. After dialyzed extensively against PBS buffer for 30 min at room temperature, the slides were incubated with 50 μl TUNEL reaction mixture covered with parafilm pieces for 60 min at 37°C in a humidified atmosphere in the dark. To identify cell types undergoing apoptosis, double staining was performed by combining TUNEL and immunohistochemistry for CD68 (macrophage marker). TUNEL staining sections were then viewed using inverted fluorescent microscope and take photography. Only

TUNEL-positive cells that colocalized with DAPI-stained nuclei were counted as positive and three sections of each mouse were quantified to detect the apoptotic cell number.

In vivo foam cell formation

In vivo foam cell formation was performed as previously described.(6) Briefly, *Ldlr*$^{-/-}$ mice transplanted with WT or *Setdb2*^{GT} BM cells were fed with WD for 12 weeks in an IACUC-approved animal facility. Peritoneal cells were collected from the peritoneal cavity 4 days after i.p. administration of 3% thioglycollate medium and allowed to adhere for 30 min. After removing non-adherent cells, the adherent macrophages were stained with Oil-Red O for foam cell formation analysis.

Collection of human specimens

Human left main coronary arteries were obtained from the explanted hearts of transplant recipients or cadaver organ donors.(7) Research protocols were approved by the Institutional Review Boards of Yale University and the New England Organ Bank. A waiver for consent was approved for surgical patients and written informed consent was obtained from a member of the family for deceased organ donors. Progressive stage of atherosclerotic plaques (No/mild and Severe stage) were distinguished through morphological characteristics of H&E staining.

Ex vivo foam cell formation

Bone marrow-derived macrophages (BMDM) from WT and *Setdb2*^{GT} mice plated in 12-well plates with cover glass were exposure to acetylated LDL (acLDL,120 μg/ml) for 24 hrs in the presence of 10% lipoprotein deficient serum (LPDS, Biomedical Technologies, Inc) and detected by Oil-Red O staining as described previously (8) The mean integrated optical density (IOD) of Oil Red O-stained region per cell was quantified with 200 representative cells using the Image J software. The cellular total cholesterol level was detected with Amplex Red Cholesterol Assay Kit (ThermoFisher), according to the manufacturer's instructions. The results were expressed as cholesterol (μg) per total cell protein (mg).

Western blot analysis

Cells were lysed in ice-cold buffer containing 50 mM Tris-HCl, pH 7.4, 0.1 mM EDTA, 0.1 mM EGTA, 1% NP-40, 0.1% sodium deoxycholate, 0.1% SDS, 100 mM NaCl, 10 mM NaF, 1 mM sodium pyrophosphate, 1 mM sodium orthovanadate, 1 mM Pefabloc, and 2 mg/ml protease inhibitor cocktail (Roche Diagnostics Corp). Protein concentrations were determined using the DC Protein assay kit (Bio-Rad Laboratories). Cell lysates containing 20 μg of protein were analyzed by SDS-PAGE and immunoblotting. Primary antibodies used include the following: anti-Setdb2 (Abcam, ab133174), anti-Nos2 (Cell Signaling, #2982) and anti-HSP90 (BD Biosciences, #610419). Secondary antibodies were fluorescence-labeled antibodies and bands were visualized using the Odyssey Infrared Imaging System (LI-COR Biotechnology).

qPCR analysis

Total RNA from cells was isolated using TRIzol reagent. One microgram of total RNA was reverse-transcribed using the iScript RT Supermix (Bio-Rad, Hercules, CA, USA), following the manufacturer's protocol. Quantitative real-time PCR was performed in triplicate using iQ SYBR green Supermix (Bio-Rad) on a Real-Time Detection System (BioRad). The mRNA level was normalized to ribosomal RNA 18S or GAPDH as a housekeeping gene. Real-time PCR was conducted with gene expression levels with oligonucleotides specific for each of the genes (**Supplemental Table 1**).

Cell culture

BMDMs from WT or *Setdb2*^{GT} mice were cultured in medium containing RPMI 1640 (Invitrogen) with 100 U penicillin/streptomycin. For M1/M2 polarization analysis, WT BMDMs were stimulated for 12 h with IL-4 (15 ng/ml, R&D, Cat 404-ML-010) and LPS (10 ng/ml, Sigma, L2630) to polarize macrophages to M2 or M1, respectively. For LPS or IFN-induced inflammation, WT and *Setdb2*^{GT} macrophages were treated with LPS (100 ng/ml) for 4 hrs or IFNγ (5 ng/ml) and IFNβ (10 ng/ml) for 12 hrs before harvest for experiment as indicated. At the end of the treatments, cells were extensively washed with 1x PBS and RNA were isolated for macrophage inflammation analysis.

Plasma cytokines measurements

Plasma cytokines including TNFα and IL-1β were measured on a BD SRII Flow Cytometer (BD Biosciences, San Jose, CA) using the LEGENDplex Mouse Inflammation Panel and LEGENDplex v.8.0 software (BioLegend, San Diego, CA).

Statistical analysis. All data are expressed as mean ± s.e.m. Statistical differences were calculated with either unpaired two-sided Student's t-test, one-way analysis of variance (ANOVA, followed by the Newman-Keuls post-test) or two-way ANOVA (followed by the Bonferroni post- test). A value of $P \leq 0.05$ was considered statistically significant. Data analysis was performed using GraphPad Prism Software Version 7.0 (GraphPad, San Diego, CA).

REFERENCES

1. Yu J, Fernandez-Hernando C, Suarez Y, Schleicher M, Hao Z, Wright PL, DiLorenzo A, Kyriakides TR, and Sessa WC. Reticulon 4B (Nogo-B) is necessary for macrophage infiltration and tissue repair. *Proc Natl Acad Sci U S A.* 2009;106(41):17511-6.

2. Goedeke L, Rotllan N, Canfran-Duque A, Aranda JF, Ramirez CM, Araldi E, Lin CS, Anderson NN, Wagschal A, de Cabo R, et al. MicroRNA-148a regulates LDL receptor and ABCA1 expression to control circulating lipoprotein levels. *Nat Med.* 2015;21(11):1280-9.

3. Zheng GX, Terry JM, Belgrader P, Ryvkin P, Bent ZW, Wilson R, Ziraldo SB, Wheeler TD, McDermott GP, Zhu J, et al. Massively parallel digital transcriptional profiling of single cells. *Nat Commun.* 2017;8(14049.

4. Satija R, Farrell JA, Gennert D, Schier AF, and Regev A. Spatial reconstruction of single-cell gene expression data. *Nat Biotechnol.* 2015;33(5):495-502.

5. Guevara NV, Kim HS, Antonova EI, and Chan L. The absence of p53 accelerates atherosclerosis by increasing cell proliferation in vivo. *Nat Med.* 1999;5(3):335-9.

6. Spann NJ, Garmire LX, McDonald JG, Myers DS, Milne SB, Shibata N, Reichart D, Fox JN, Shaked I, Heudobler D, et al. Regulated accumulation of desmosterol integrates macrophage lipid metabolism and inflammatory responses. *Cell.* 2012;151(1):138-52.

7. Chen PY, Qin L, Li G, Tellides G, and Simons M. Smooth muscle FGF/TGFbeta cross talk regulates atherosclerosis progression. *EMBO Mol Med.* 2016;8(7):712-28.

8. Canfran-Duque A, Rotllan N, Zhang X, Fernandez-Fuertes M, Ramirez-Hidalgo C, Araldi E, Daimiel L, Busto R, Fernandez-Hernando C, and Suarez Y. Macrophage deficiency of miR-21 promotes apoptosis, plaque necrosis, and vascular inflammation during atherogenesis. *EMBO Mol Med.* 2017;9(9):1244-62.

SUPPLEMENTAL FIGURE LEGENDS

Supplemental Figure 1. Setdb2 deficiency in hematopoietic cells increases body weight but does not influence plasma lipid and circulating leukocytes. A) Body weight (BW) of *Ldlr*$^{-/-}$ mice transplanted with WT or *Setdb2*^{GT} bone marrow (BM) cells fed a Western diet (WD) for 14 weeks (WT, n=8; *Setdb2*^{GT}, n=10). **B**) Analysis of plasma total cholesterol, high density lipoprotein cholesterol (HDL-C) and triglycerides from *Ldlr*$^{-/-}$ mice transplanted with WT or *Setdb2*^{GT} BM cells before and after WD feeding (WT, n=8; *Setdb2*^{GT}, n=10). **C**) Analysis of blood glucose from *Ldlr*$^{-/-}$ mice transplanted with WT or *Setdb2*^{GT} BM cells after WD feeding (WT, n=9; *Setdb2*^{GT}, n=8). **D**) Hemavet analysis of cellular composition from peripheral blood of *Ldlr*$^{-/-}$ mice transplanted with WT or *Setdb2*^{GT} BM cells before and after WD feeding. **E and F**) Flow cytometry analysis of Ly6C^{hi} and Ly6C^{lo} monocytes, neutrophils, B Cells and CD4^{+} and CD8^{+} lymphocytes from peripheral blood of *Ldlr*$^{-/-}$ mice transplanted with WT or *Setdb2*^{GT} BM cells before (**E**) and after WD feeding (**F**). Quantification represents the mean ± S.E.M. * P<0.05 compared to *Ldlr*$^{-/-}$ mice transplanted with WT BM. Data were analyzed by one-way ANOVA and Tukey's post hoc test (**B**) or unpaired two-sided Student’s t-test (**C-F**).

Supplemental Figure 2. Hematopoietic Setdb2 deficiency does not affect neutral lipid accumulation in the lesions neither macrophage foam cell formation in vivo. A) Representative histological analysis of cross-sections of the aortic sinus stained with Oil Red O in *Ldlr*$^{-/-}$ mice transplanted with WT or *Setdb2*^{GT} BM fed a Western diet (WD) for 14 weeks. Mean of the % of Oil Red O positive area vs total plaque is shown in the right panel (n=8-10 mice per group). **B**) Representative Oil Red O staining of thioglycolate-elicited foamy macrophages from *Ldlr*$^{-/-}$ mice transplanted with WT or *Setdb2*^{GT} BM fed a Western diet (WD) for 12 weeks (n=4 per group). Quantification of the Oil red O positive area and the integrated optical density (IDO) per cell is shown in right panels. Data represents the mean ± S.E.M. of the quantification of four images from different fields for each peritoneal macrophage isolation. **C**) Representative ORO staining in bone marrow derived macrophages (BMDMs) isolated from WT or

Setdb2^{GT} mice incubated with acLDL (120 μg/ml) for 24 hrs (n=4 per group). Quantification represents the mean ± s.e.m. Data were analyzed by an unpaired two-sided Student's t-test.

Supplemental Figure 3. Single cell RNA-Seq analysis on CD45^{+} cells isolated from atherosclerotic plaques transplanted with *Setdb2*^{GT} Bone marrow (BM) cells. Bone marrow (BM) cells isolated from WT and *Setdb2*^{GT} mice were transplanted to lethally radiated *Ldlr*$^{-/-}$ mice and fed a western diet (WD) for 16 weeks to induce atherosclerosis. Atherosclerotic aortas were enzymatically digested and CD45^{+} cells were isolated by flow cytometry. Feature plots depicting single-cell gene expression of individual genes.

Supplemental Figure 4. Single cell RNA-Seq analysis reveals increased monocyte and neutrophil infiltration in atherosclerotic plaques transplanted with *Setdb2*^{GT} Bone marrow (BM) cells. A) Distribution of defined cell populations from the atherosclerotic aortas of *Ldlr*$^{-/-}$ mice transplanted with WT or *Setdb2*^{GT} BM. **B**) Violin plots of the top differentially expressed transcripts showing statistically significant upregulation in CD45^{+} cells isolated from *Ldlr*$^{-/-}$ mice transplanted with WT and *Setdb2*^{GT} bone marrow cells.

Supplemental Figure 5. Effects of *Setdb2* deficiency in haematopoietic cells on macrophage apoptosis and efferocytosis in atherosclerotic lesions. A) Representative analysis of macrophage apoptosis by Terminal deoxynucleotidyl transferase dUTP nick end labeling (TUNEL) in atherosclerotic lesions of the aortic valve from WT and *Setdb2*^{GT} BM transplanted mice (n=6 each group). **B**) Representative immunofluorescence staining of MerTK expression in atherosclerotic lesions of the arotic valve from WT and *Setdb2*^{GT} BM transplanted mice (n=7 each group). Data were analyzed by an unpaired two-sided Student's t-test. Quantification represents the mean ± s.e.m.

Supplemental Figure 6. Deficiency of SETDB2 in haematopoietic cells impairs macrophage proliferation in atherosclerotic plaques. Representative immunofluorescence analysis of aortic root cross-sections of the aortic root lesions stained with Ki67 (proliferation marker) and CD68 (*macrophage*

marker) in *Ldlr*$^{-/-}$ mice transplanted with WT or *Setdb2*^{GT} BM fed a Western diet (WD) for 14 weeks. Representative Dashed lines indicate the atherosclerotic lesion. Quantifications are shown in the right panel (n=7-9 mice per group). Data were analyzed by an unpaired two-sided Student's t-test. Quantification represents the mean ± s.e.m.

Supplemental Figure 1.

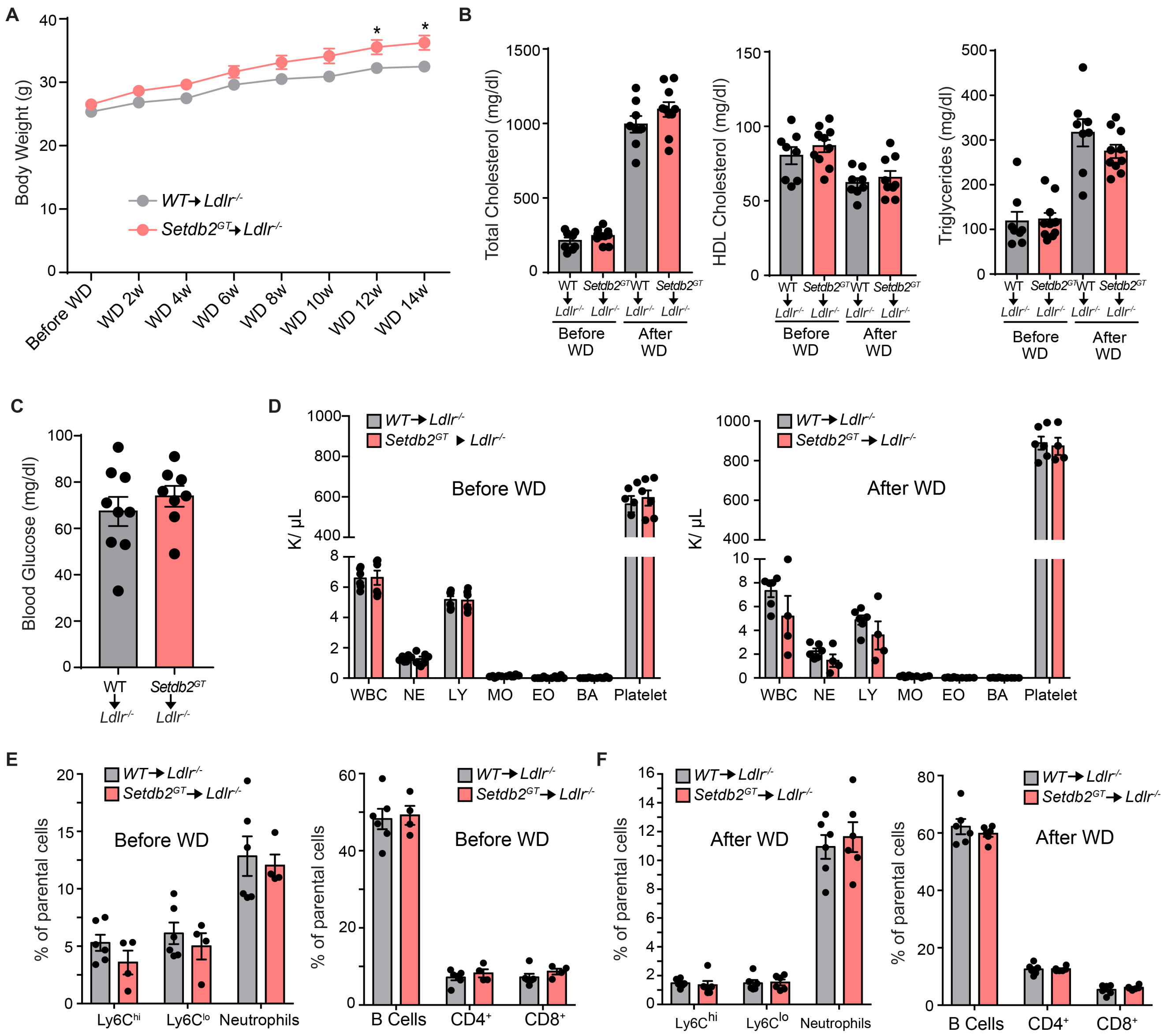

Supplemental Figure 2.

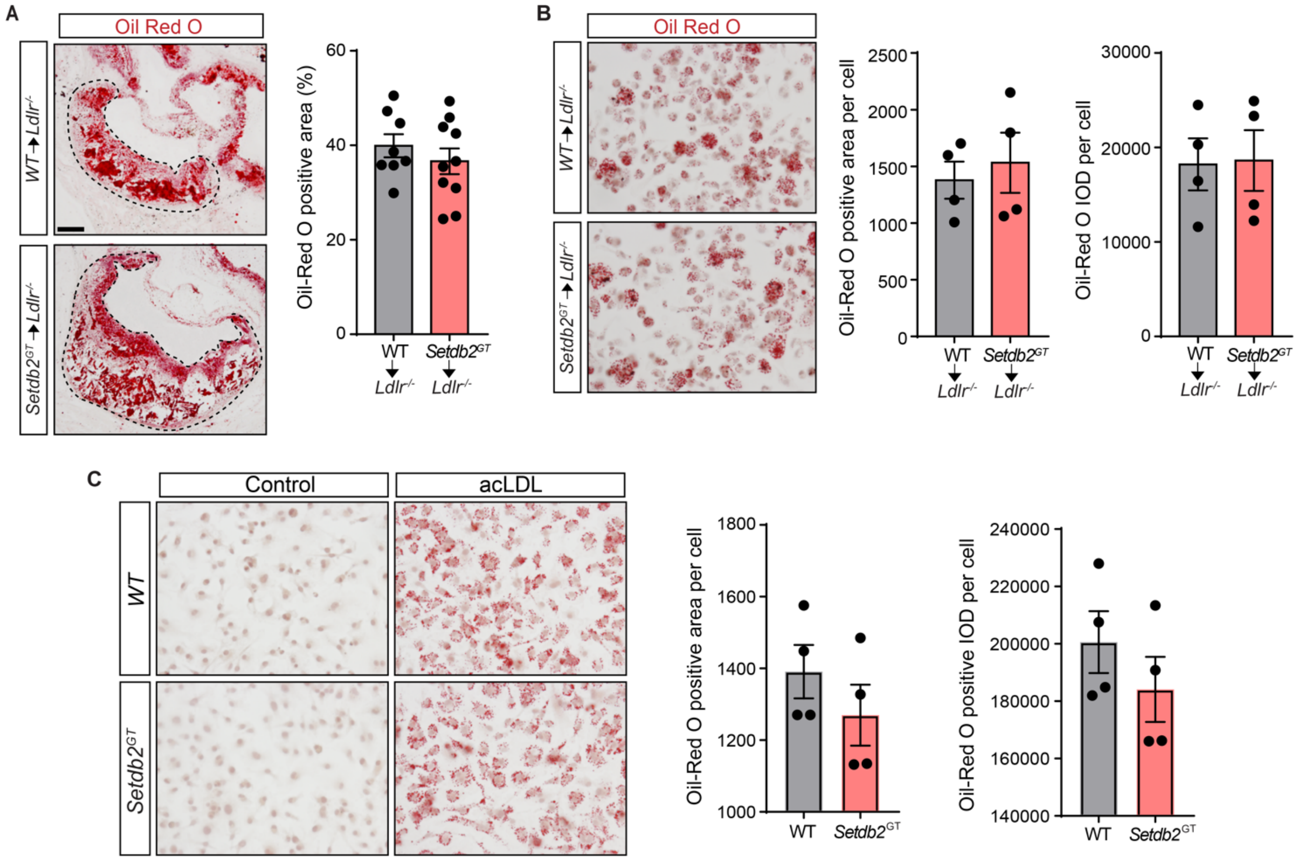

Supplemental Figure 3.

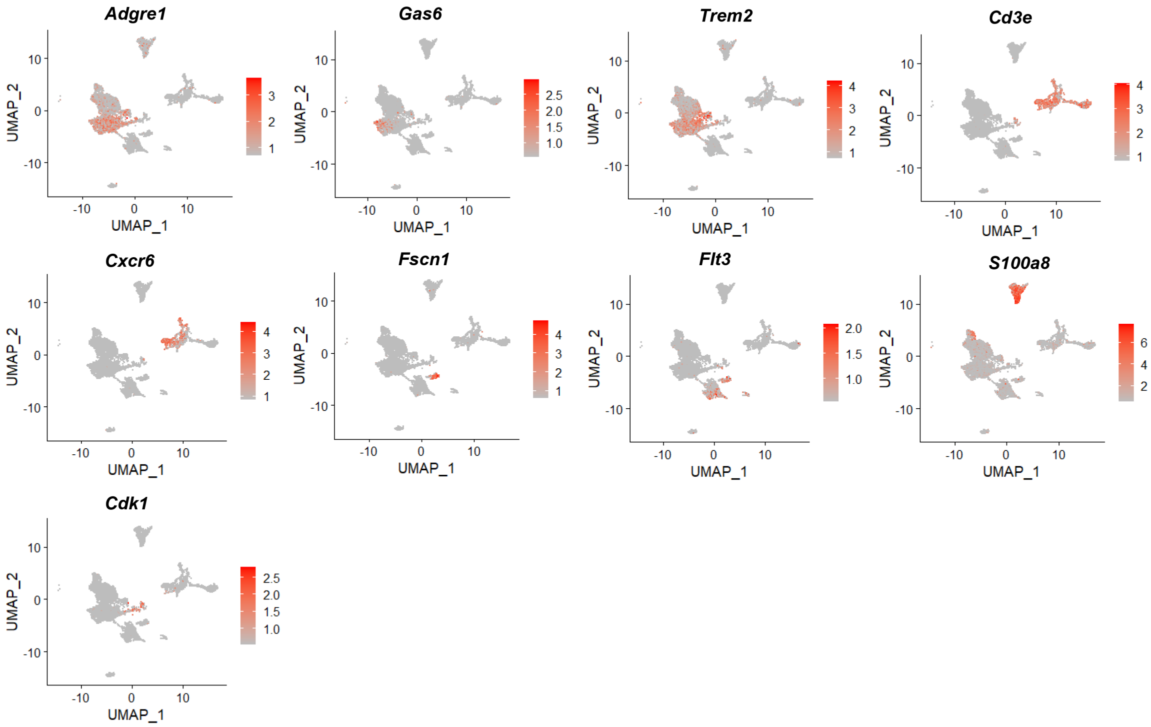

Supplemental Figure 4.

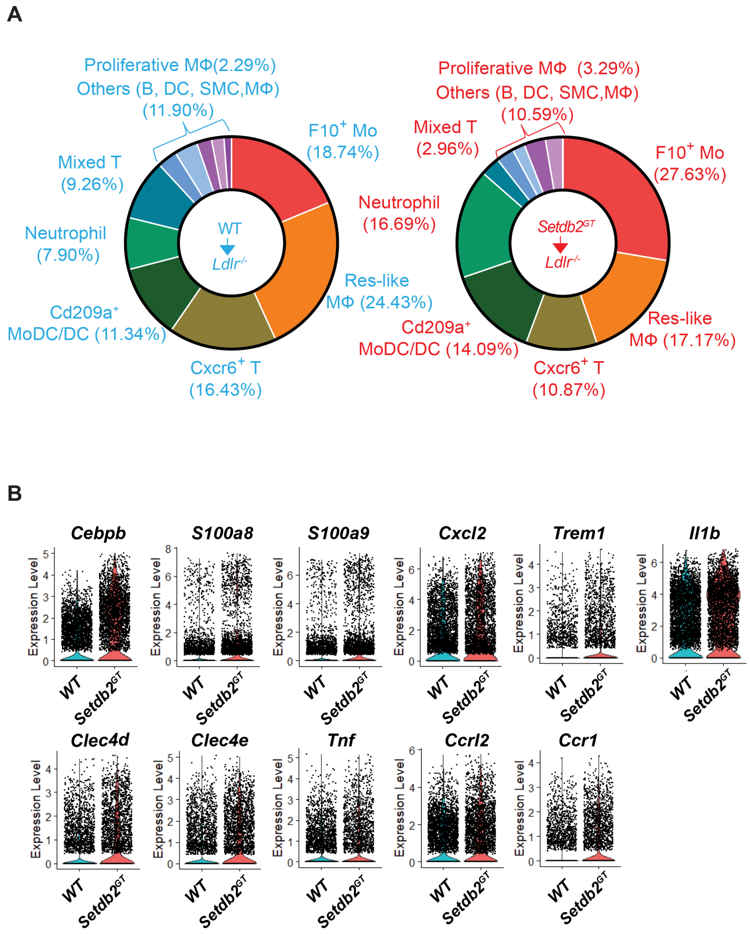

Supplemental Figure 5.

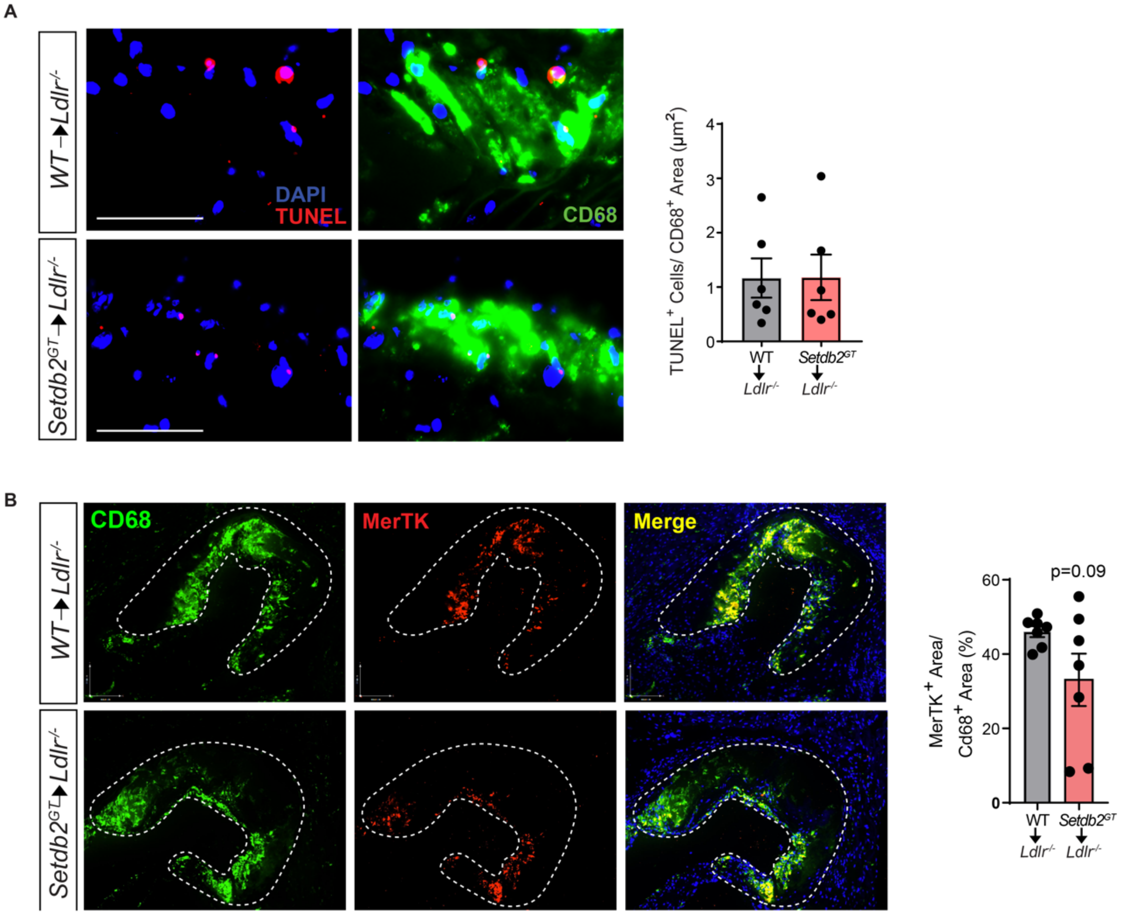

Supplemental Figure 6.

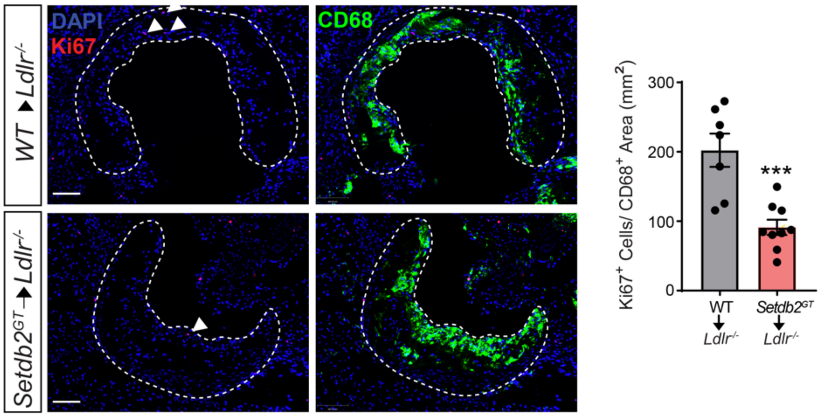

Supplemental Table 1. Forward and reverse primers used for RT-PCR.

Name	Species	Primers	Sequence (5' to 3')
Setdb2	Mouse	Forward	GGATGGAGCTACAAGATGATGG
		Reverse	CCAGTGTTTGCGTGTTACTCAG
SETDB2	Human	Forward	TGCTCTGGTGCTTGTCTGATG
		Reverse	TGCATGTCGTCTTTGGAAGTG
IL-6	Mouse	Forward	TAGTCCTTCCTACCCCAATTTCC
		Reverse	TTGGTCCTTAGCCACTCCTTC
Ccl2	Mouse	Forward	TTAAAAACCTGGATCGGAACCAA
		Reverse	GCATTAGCTTCAGATTTACGGGT
IL-10	Mouse	Forward	GCTCTTACTGACTGGCATGAG
		Reverse	CGCAGCTCTAGGAGCATGTG

Supplemental Table 2. Differentially upregulated genes in CD45$^+$ leukocytes isolated from atherosclerotic plaques of *Ldlr*$^{-/-}$ mice transplanted with WT or *Setdb2*^{GT} BM.

Gene	p_value	avg_logFC	Setdb2^{GT}	WT	p_val_adj
Cebpb	2.35E-222	1.1417275	0.726	0.506	2.69E-218
S100a8	1.84E-141	0.9683975	0.556	0.298	2.11E-137
Hebp1	3.97E-102	0.3437911	0.301	0.127	4.55E-98
S100a9	1.57E-95	0.7850869	0.519	0.31	1.80E-91
Clec4d	3.88E-74	0.5486197	0.461	0.29	4.45E-70
Emp1	2.32E-68	0.3671773	0.328	0.178	2.65E-64
S100a6	2.43E-66	0.3667722	0.845	0.753	2.79E-62
Clec4e	5.05E-66	0.5254145	0.469	0.304	5.79E-62
Chil3	6.86E-63	0.8985752	0.265	0.131	7.86E-59
Tyrobp	2.55E-60	0.2788778	0.889	0.813	2.92E-56
S100a11	2.72E-59	0.427675	0.872	0.799	3.12E-55
Trem1	5.89E-59	0.5606838	0.315	0.175	6.75E-55
Gsr	7.27E-56	0.3590206	0.49	0.335	8.33E-52
Msrb1	4.61E-55	0.4236054	0.723	0.599	5.28E-51
Saa3	3.13E-54	0.5311535	0.103	0.026	3.59E-50
Ccr1	1.30E-53	0.3907416	0.379	0.236	1.50E-49
Slpi	2.59E-53	0.5461142	0.323	0.185	2.97E-49
Cd44	4.58E-49	0.2973388	0.623	0.487	5.25E-45
Srxn1	1.51E-48	0.2725104	0.216	0.107	1.73E-44
Fth1	5.17E-47	0.3720405	0.998	0.996	5.92E-43
Ier3	1.49E-46	0.5229755	0.561	0.43	1.71E-42
Wfdc17	3.27E-46	0.5010249	0.748	0.649	3.74E-42
Ifitm2	1.31E-45	0.311547	0.843	0.761	1.50E-41
Card19	4.89E-43	0.300098	0.635	0.513	5.60E-39
Pkm	6.24E-42	0.2823325	0.863	0.808	7.15E-38
Rnf149	5.97E-41	0.3093586	0.618	0.497	6.84E-37
Srgn	1.11E-40	0.3253647	0.929	0.902	1.27E-36
Cstb	2.70E-40	0.4184389	0.797	0.724	3.09E-36
Lilrb4a	2.28E-38	0.2668418	0.656	0.548	2.61E-34
Alox5ap	4.65E-37	0.2570126	0.738	0.643	5.33E-33

Clec4n	4.69E-37	0.4057903	0.445	0.329	5.37E-33
Plaur	3.09E-36	0.3008976	0.47	0.35	3.54E-32
Irg1	4.42E-36	0.4862101	0.184	0.096	5.06E-32
Ifitm1	1.37E-35	0.4743144	0.481	0.363	1.58E-31
Cd9	9.04E-34	0.5049917	0.561	0.454	1.04E-29
Arg2	9.18E-34	0.344633	0.219	0.127	1.05E-29
Lgals3	1.68E-33	0.2524166	0.837	0.779	1.93E-29
Ninj1	1.29E-32	0.3014651	0.662	0.552	1.47E-28
Il1r2	1.96E-32	0.445355	0.396	0.288	2.25E-28
Slc16a3	6.73E-32	0.3763938	0.354	0.25	7.71E-28
Hcar2	1.95E-31	0.535057	0.197	0.112	2.24E-27
Hp	1.99E-31	0.3698355	0.295	0.195	2.29E-27
Pla2g7	2.08E-31	0.3301588	0.445	0.347	2.38E-27
Sirpb1c	7.04E-30	0.2917764	0.37	0.27	8.07E-26
Slc7a11	8.00E-30	0.2914294	0.317	0.213	9.17E-26
Mcemp1	1.14E-29	0.2858785	0.353	0.246	1.31E-25
Vim	3.59E-29	0.2773965	0.805	0.765	4.11E-25
Retnlg	4.73E-29	0.6653717	0.104	0.044	5.42E-25
Ftl1	1.76E-28	0.2510577	0.993	0.991	2.02E-24
Hmox1	1.65E-27	0.4530366	0.334	0.241	1.90E-23
Cd14	9.95E-27	0.3349988	0.683	0.595	1.14E-22
Hdc	1.84E-26	0.3879052	0.16	0.088	2.11E-22
Tnfaip2	2.03E-25	0.2630582	0.377	0.278	2.33E-21
Grina	8.12E-25	0.4111467	0.542	0.441	9.30E-21
Basp1	2.04E-23	0.2510378	0.442	0.356	2.34E-19
Il1b	2.07E-23	0.3563554	0.825	0.774	2.38E-19
Samsn1	3.23E-23	0.2906127	0.428	0.334	3.70E-19
Cxcl2	1.49E-21	0.6824424	0.69	0.605	1.71E-17
C5ar1	4.85E-21	0.2755375	0.518	0.439	5.56E-17
Lmnb1	6.62E-21	0.3186104	0.532	0.444	7.59E-17
Prdx5	4.45E-20	0.2599142	0.805	0.75	5.10E-16
Csf3r	1.59E-18	0.2971536	0.233	0.163	1.82E-14
G0s2	5.95E-18	0.3502874	0.151	0.093	6.82E-14
Nfkbia	4.78E-16	0.3505359	0.82	0.769	5.48E-12

Klf2	9.66E-16	0.3998912	0.635	0.561	1.11E-11
Spp1	3.20E-14	0.4838331	0.454	0.381	3.67E-10
Mxd1	1.50E-12	0.3355986	0.366	0.304	1.72E-08
Il1rn	1.82E-11	0.2739472	0.429	0.37	2.09E-07
Hilpda	4.49E-11	0.3359746	0.49	0.433	5.15E-07
Ccl6	1.95E-10	0.2506623	0.623	0.568	2.24E-06
Txnip	2.64E-10	0.2770144	0.351	0.295	3.03E-06
Ctsd	8.38E-10	0.2602852	0.812	0.776	9.60E-06
Il1a	4.71E-08	0.3810022	0.12	0.087	5.40E-04
AA467197	1.00E-07	0.2991285	0.4	0.353	1.15E-03
Tnf	1.81E-06	0.4235783	0.349	0.312	2.07E-02

Supplemental Table 3. Differentially downregulated genes in CD45$^+$ leukocytes isolated from atherosclerotic plaques of *Ldlr*$^{-/-}$ mice transplanted with WT or *Setdb2*^{GT} BM.

Gene	p_value	avg_logFC	Setdb2^{GT}	WT	p_val_adj
Mgp	8.56E-120	-1.2671974	0.05	0.221	9.82E-116
Ccl5	7.64E-83	-0.70189	0.301	0.491	8.76E-79
Cd3d	6.96E-76	-0.5663933	0.157	0.325	7.97E-72
Cd3g	6.23E-70	-0.5900934	0.158	0.316	7.14E-66
AY036118	3.11E-66	-0.2799334	0.184	0.328	3.56E-62
Cd8b1	1.71E-65	-0.6574216	0.098	0.233	1.96E-61
Nkg7	1.11E-60	-0.5987617	0.11	0.246	1.27E-56
Lck	2.80E-60	-0.5361366	0.137	0.275	3.21E-56
Cd8a	9.34E-59	-0.5321312	0.089	0.211	1.07E-54
AW112010	2.13E-55	-0.4829127	0.443	0.573	2.44E-51
Cd3e	1.30E-53	-0.4103892	0.129	0.26	1.49E-49
Cd27	2.07E-53	-0.5294914	0.085	0.197	2.38E-49
H2-Q7	2.97E-53	-0.3633819	0.392	0.532	3.40E-49
Ccl8	6.56E-53	-0.3964676	0.128	0.253	7.52E-49
Ly6a	8.05E-53	-0.4452079	0.32	0.461	9.22E-49
Thy1	9.50E-52	-0.4573291	0.141	0.27	1.09E-47
Lat	6.97E-51	-0.4682001	0.15	0.278	7.99E-47
Uba52	7.48E-48	-0.2665328	0.6	0.676	8.57E-44
Cd247	9.27E-43	-0.4055928	0.082	0.178	1.06E-38
C1qb	1.01E-40	-0.2709775	0.59	0.709	1.16E-36
1-Sep	8.23E-40	-0.3907729	0.149	0.257	9.43E-36
Sh2d1a	1.31E-38	-0.3329611	0.058	0.141	1.50E-34
Ptprcap	4.90E-37	-0.3833437	0.175	0.283	5.61E-33
Gimap6	1.51E-35	-0.3427918	0.13	0.23	1.73E-31
Sh2d2a	1.75E-34	-0.3192763	0.084	0.17	2.01E-30
C1qa	3.06E-34	-0.2830074	0.543	0.654	3.51E-30
Sla2	2.90E-33	-0.3613932	0.063	0.139	3.32E-29
C1qc	1.20E-29	-0.2716765	0.49	0.601	1.37E-25
Skap1	1.96E-29	-0.3079372	0.103	0.185	2.24E-25
Limd2	5.19E-28	-0.264179	0.621	0.685	5.95E-24

Tcf7	1.19E-26	-0.3690582	0.067	0.134	1.36E-22
Gimap1	1.59E-26	-0.275282	0.097	0.174	1.82E-22
Gimap4	2.49E-26	-0.3059098	0.107	0.186	2.86E-22
Ms4a4b	4.95E-26	-0.4547811	0.142	0.223	5.67E-22
Ets1	7.07E-26	-0.2668345	0.113	0.192	8.11E-22
Cd28	1.64E-25	-0.3408403	0.094	0.167	1.88E-21
Ifi27l2a	2.07E-25	-0.2821137	0.719	0.771	2.37E-21
Sox4	3.66E-25	-0.4905753	0.066	0.13	4.19E-21
Rhoh	5.96E-24	-0.3489253	0.223	0.31	6.84E-20
H2afv	2.43E-23	-0.321561	0.411	0.478	2.79E-19
Ccl7	5.40E-23	-0.2605236	0.198	0.285	6.18E-19
Satb1	2.18E-20	-0.5298851	0.104	0.167	2.49E-16
Tubb5	2.26E-20	-0.2924145	0.564	0.623	2.59E-16
Cd72	9.56E-20	-0.2568828	0.351	0.431	1.10E-15
Ldhb	1.22E-18	-0.4802465	0.082	0.137	1.40E-14
Hist3h2ba	1.19E-17	-0.3454857	0.064	0.115	1.36E-13
Tubb2b	4.75E-16	-0.2837646	0.059	0.105	5.44E-12
Spint2	9.41E-16	-0.3468689	0.152	0.211	1.08E-11
Dusp10	1.52E-15	-0.2800979	0.101	0.153	1.75E-11
Stmn1	1.25E-14	-0.3876775	0.168	0.225	1.43E-10
Gramd3	5.00E-14	-0.3325104	0.126	0.179	5.73E-10
Tubb2a	1.00E-12	-0.3031586	0.304	0.357	1.15E-08
Mier1	1.90E-11	-0.4055223	0.254	0.3	2.18E-07
Hmgn1	4.12E-11	-0.2569348	0.346	0.395	4.73E-07
Ssbp2	4.77E-10	-0.3448256	0.131	0.171	5.47E-06
Rnf157	2.20E-07	-0.2899112	0.096	0.127	2.52E-03
Hmgb1	1.18E-06	-0.2596131	0.747	0.755	1.36E-02

Supplemental Table 4. Differentially upregulated genes in monocytes/macrophages population isolated from atherosclerotic plaques of *Ldlr*$^{-/-}$ mice transplanted with WT or *Setdb2*^{GT} BM.

Gene	p_value	avg_logFC	Setdb2^{GT}	WT	p_val_adj
Cebpb	1.15E-185	1.29448237	0.913	0.767	1.40E-181
Hebp1	3.73E-90	0.662786799	0.503	0.227	4.53E-86
Rps28	2.27E-87	0.384492685	0.989	0.984	2.75E-83
Rpl38	8.09E-82	0.376276998	0.991	0.987	9.81E-78
Rps27	4.46E-78	0.35489264	0.997	0.995	5.40E-74
Rps29	5.03E-70	0.302143227	0.998	0.999	6.10E-66
S100a8	2.32E-66	0.925720582	0.554	0.31	2.81E-62
Rpl41	1.44E-64	0.294692237	1	0.999	1.75E-60
Emp1	9.28E-60	0.712123302	0.504	0.279	1.12E-55
Chil3	8.29E-56	1.515852236	0.453	0.242	1.01E-51
Rpl37a	3.17E-53	0.267423291	1	0.997	3.85E-49
Hspa8	3.79E-49	0.378263314	0.994	0.993	4.60E-45
Rpl36	9.11E-41	0.257519437	0.987	0.988	1.10E-36
Sec61g	1.08E-40	0.375877565	0.945	0.916	1.31E-36
Rbm3	3.99E-40	0.350327911	0.947	0.939	4.84E-36
Rpl39	5.15E-39	0.250017362	0.996	0.995	6.24E-35
S100a6	8.41E-38	0.554415368	0.89	0.836	1.02E-33
S100a11	1.66E-33	0.564821958	0.894	0.846	2.01E-29
S100a9	6.19E-32	0.670274808	0.495	0.325	7.50E-28
Dynll1	2.16E-31	0.308490153	0.952	0.929	2.62E-27
Clec4e	1.12E-30	0.488798658	0.598	0.434	1.36E-26
2010107E04Rik	7.67E-29	0.269234558	0.912	0.854	9.30E-25
Pla2g7	1.03E-28	0.480783452	0.728	0.614	1.25E-24
Srxn1	1.24E-28	0.324161617	0.312	0.169	1.50E-24
Clec4d	6.27E-28	0.469305238	0.582	0.423	7.60E-24
S100a4	1.87E-27	0.463839111	0.806	0.724	2.27E-23
Vim	2.39E-27	0.437116391	0.951	0.937	2.90E-23
Gsr	3.07E-27	0.438511184	0.576	0.421	3.72E-23
Pkm	6.82E-26	0.380552822	0.945	0.937	8.26E-22
Slpi	1.91E-24	0.63169251	0.372	0.231	2.31E-20

F10	5.02E-24	0.379510489	0.432	0.282	6.09E-20
Lilrb4a	1.52E-21	0.31201669	0.883	0.819	1.84E-17
Clec4n	4.92E-21	0.497452205	0.589	0.471	5.96E-17
Cstb	5.01E-21	0.37348707	0.948	0.933	6.08E-17
Cd44	3.95E-20	0.29941383	0.759	0.655	4.79E-16
Hmox1	2.28E-19	0.758481272	0.542	0.424	2.77E-15
Ccr1	3.33E-19	0.3488256	0.43	0.302	4.04E-15
Napsa	4.69E-19	0.359418971	0.639	0.522	5.69E-15
Usmg5	6.00E-19	0.252246179	0.826	0.746	7.27E-15
Hspa5	7.42E-19	0.334332984	0.908	0.887	9.00E-15
Hp	1.03E-18	0.566605415	0.399	0.28	1.25E-14
Slc7a11	4.91E-18	0.406656964	0.351	0.235	5.96E-14
Adssl1	5.19E-18	0.258928256	0.513	0.385	6.29E-14
Emb	7.25E-18	0.341890586	0.6	0.489	8.79E-14
Aldoa	1.76E-17	0.302543325	0.921	0.886	2.13E-13
Trem1	3.11E-17	0.312859866	0.321	0.209	3.77E-13
Mgst1	9.30E-17	0.330516197	0.395	0.275	1.13E-12
Emilin2	3.24E-16	0.290806857	0.542	0.435	3.93E-12
Txnrd1	4.03E-16	0.267403564	0.518	0.402	4.89E-12
Apobec1	4.24E-16	0.30217997	0.659	0.567	5.14E-12
Msrb1	5.91E-16	0.300543412	0.83	0.784	7.16E-12
Plaur	6.24E-16	0.27894757	0.638	0.523	7.56E-12
Sirpb1c	7.13E-16	0.341491147	0.548	0.441	8.65E-12
Lgals3	1.30E-15	0.278647608	0.982	0.972	1.57E-11
Ier3	2.63E-14	0.479823638	0.703	0.615	3.19E-10
S100a10	4.42E-14	0.290701759	0.814	0.777	5.35E-10
Msr1	6.53E-14	0.256104465	0.434	0.329	7.92E-10
Adam8	7.21E-14	0.269501194	0.329	0.232	8.74E-10
Rnh1	1.40E-13	0.275327664	0.84	0.785	1.69E-09
Txn1	4.73E-13	0.250197603	0.939	0.922	5.74E-09
Tgfbi	8.80E-13	0.275238019	0.914	0.893	1.07E-08
Anxa1	6.40E-12	0.280017246	0.661	0.582	7.76E-08
Por	6.90E-12	0.250702549	0.418	0.323	8.37E-08
Slc16a3	2.08E-11	0.290200739	0.45	0.357	2.52E-07

Prdx6	4.75E-11	0.282677454	0.767	0.704	5.75E-07
Basp1	1.85E-10	0.277748278	0.587	0.521	2.24E-06
Ctsl	2.62E-10	0.442474355	0.78	0.737	3.18E-06
Prdx5	8.92E-10	0.269259576	0.928	0.917	1.08E-05
Rgcc	9.90E-10	0.314768594	0.301	0.221	1.20E-05
Esd	1.64E-08	0.261672688	0.813	0.784	0.000198264
Ifitm1	3.14E-08	0.533994663	0.457	0.382	0.000381075
Tmsb10	3.83E-08	0.258015283	0.9	0.909	0.00046416
Thbs1	1.90E-07	0.377932093	0.48	0.418	0.002299366
Ctsd	4.24E-07	0.279271225	0.934	0.923	0.00513905
Plac8	8.07E-07	0.26049939	0.598	0.54	0.009786754

Supplemental Table 5. Differentially downregulated genes in monocytes/macrophages population isolated from atherosclerotic plaques of *Ldlr*$^{-/-}$ mice transplanted with WT or *Setdb2*^{GT} BM.

Gene	p_value	avg_logFC	Setdb2^{GT}	WT	p_val_adj
Mgp	1.02E-69	-0.36097	0.074	0.292	1.23E-65
Ccl5	3.12E-52	-0.33506	0.305	0.519	3.78E-48
Gm26917	5.05E-48	-0.33598	0.29	0.481	6.13E-44
Ly6a	4.20E-45	-0.8153	0.345	0.54	5.09E-41
AY036118	1.85E-44	-0.34473	0.22	0.407	2.24E-40
Ccl8	1.84E-43	-0.64911	0.178	0.369	2.23E-39
AW112010	2.57E-36	-0.54878	0.517	0.669	3.11E-32
H2-Ab1	4.86E-34	-0.40997	0.964	0.987	5.89E-30
C1qb	9.75E-33	-0.43652	0.809	0.912	1.18E-28
Aif1	6.24E-32	-0.36998	0.81	0.873	7.56E-28
H2-Eb1	2.07E-31	-0.39129	0.916	0.965	2.51E-27
Ccl4	2.69E-31	-0.52054	0.553	0.696	3.27E-27
C1qa	3.76E-31	-0.44638	0.752	0.873	4.56E-27
Ly86	1.56E-30	-0.37259	0.797	0.852	1.89E-26
Cd74	5.67E-29	-0.37598	0.994	0.998	6.87E-25
Psmb8	5.87E-29	-0.2865	0.902	0.934	7.11E-25
Uba52	6.00E-29	-0.30458	0.709	0.786	7.27E-25
H2-Aa	9.09E-29	-0.37225	0.949	0.974	1.10E-24
Bst2	1.12E-28	-0.41951	0.769	0.834	1.36E-24
H2-Q7	2.66E-28	-0.42436	0.405	0.547	3.22E-24
C1qc	2.81E-28	-0.43511	0.726	0.842	3.41E-24
Gm42418	4.04E-27	-0.27601	0.432	0.564	4.90E-23
H2-K1	2.45E-26	-0.27064	0.962	0.969	2.97E-22
Psmb9	3.54E-24	-0.29123	0.571	0.685	4.29E-20
H2-DMb1	3.74E-23	-0.35376	0.733	0.815	4.54E-19
H2-T23	2.83E-21	-0.29877	0.422	0.537	3.43E-17
Cd72	5.10E-21	-0.40441	0.496	0.631	6.19E-17
Ly6e	2.23E-20	-0.32215	0.923	0.96	2.70E-16
Itm2b	4.31E-20	-0.28262	0.991	0.994	5.23E-16
H2-DMa	1.33E-19	-0.27624	0.804	0.856	1.62E-15

Fcgrt	5.22E-19	-0.366	0.419	0.533	6.33E-15
Sepp1	2.70E-18	-0.40266	0.832	0.861	3.28E-14
Slamf9	3.29E-18	-0.28737	0.479	0.612	3.98E-14
Ifi27l2a	6.38E-18	-0.3489	0.903	0.942	7.74E-14
Lair1	1.02E-17	-0.29238	0.508	0.608	1.24E-13
Ccl7	2.47E-17	-0.35253	0.315	0.443	3.00E-13
Ccl12	7.92E-17	-0.32525	0.323	0.451	9.61E-13
Irf7	1.69E-16	-0.32927	0.498	0.592	2.05E-12
Ms4a7	3.97E-16	-0.33974	0.649	0.75	4.81E-12
Egr1	6.31E-16	-0.41284	0.288	0.393	7.65E-12
Cd83	9.81E-16	-0.28509	0.783	0.838	1.19E-11
Nrp1	1.65E-15	-0.2796	0.32	0.422	1.99E-11
Fgl2	2.37E-15	-0.28012	0.343	0.446	2.87E-11
H2-T22	3.09E-14	-0.25872	0.436	0.527	3.75E-10
C3ar1	1.03E-13	-0.26016	0.57	0.661	1.24E-09
H2afv	3.24E-13	-0.4621	0.436	0.518	3.92E-09
Nfkbiz	4.88E-13	-0.31537	0.592	0.666	5.91E-09
Igf1	1.42E-12	-0.28253	0.26	0.364	1.72E-08
Rhoh	2.00E-12	-0.26124	0.182	0.267	2.42E-08
Mrc1	2.12E-12	-0.27181	0.233	0.322	2.57E-08
Tubb5	4.04E-09	-0.42499	0.66	0.712	4.89E-05
Apoe	9.13E-09	-0.2548	0.993	0.996	0.000111
Dek	6.51E-08	-0.2591	0.558	0.59	0.000789
Hmgn2	7.43E-08	-0.42602	0.351	0.411	0.000901
Stmn1	3.20E-07	-0.69678	0.213	0.271	0.003874

Supplemental Table 6. Differentially upregulated genes in monocyte population isolated from atherosclerotic plaques of *Ldlr*$^{-/-}$ mice transplanted with WT or *Setdb2*^{GT} BM.

Gene	p_value	avg_logFC	Setdb2^{GT}	WT	p_val_adj
Cebpb	########	1.158299	0.991	0.904	########
Hebp1	1.50E-70	0.846038	0.53	0.178	1.81E-66
Rbm3	1.96E-51	0.451796	0.987	0.976	2.38E-47
Rps28	5.52E-51	0.377105	0.997	0.996	6.70E-47
Rps29	2.27E-46	0.322906	1	1	2.75E-42
Rpl38	2.82E-45	0.370005	0.999	0.998	3.42E-41
Rps27	5.53E-40	0.32311	0.999	0.999	6.70E-36
S100a8	2.41E-39	0.963261	0.59	0.31	2.92E-35
Rpl41	3.34E-38	0.279485	1	0.999	4.05E-34
Emp1	4.40E-35	0.696233	0.622	0.364	5.33E-31
Chil3	7.84E-29	1.209997	0.682	0.481	9.50E-25
Rpl37a	2.74E-26	0.257768	1	0.999	3.32E-22
Hspa8	2.98E-25	0.38747	1	0.996	3.61E-21
Rpl39	1.94E-22	0.259477	0.998	0.996	2.35E-18
Srxn1	1.27E-21	0.386524	0.372	0.184	1.54E-17
S100a9	8.01E-21	0.691237	0.535	0.324	9.71E-17
Pla2g7	6.96E-16	0.426916	0.833	0.723	8.44E-12
Dnaja1	1.61E-14	0.30031	0.821	0.702	1.95E-10
Dynll1	5.35E-13	0.250269	0.973	0.942	6.48E-09
Hmox1	9.42E-12	0.740875	0.601	0.467	1.14E-07
S100a11	1.18E-11	0.335087	0.976	0.961	1.43E-07
Txn1	7.98E-10	0.25216	0.966	0.947	9.68E-06
Jund	1.11E-09	0.291207	0.919	0.875	1.35E-05
Rgcc	1.89E-09	0.416895	0.422	0.297	2.29E-05
Ctsl	2.02E-09	0.519945	0.806	0.708	2.45E-05
Apobec1	4.39E-09	0.266127	0.66	0.539	5.32E-05
Clec4d	5.87E-09	0.295062	0.744	0.617	7.11E-05
Hspa5	1.29E-08	0.301993	0.939	0.908	0.000156
Slc7a11	6.64E-08	0.290709	0.525	0.401	0.000804
Basp1	1.24E-07	0.312319	0.521	0.423	0.001505

Slpi	1.69E-07	0.333905	0.54	0.416	0.002051
Wfdc17	3.55E-07	0.324212	0.951	0.929	0.004306
Ier3	1.22E-06	0.378518	0.785	0.706	0.014749

Supplemental Table 7. Differentially downregulated genes in monocyte population isolated from atherosclerotic plaques of *Ldlr*$^{-/-}$ mice transplanted with WT or *Setdb2*^{GT} BM.

Gene	p_value	avg_logFC	Setdb2^{GT}	WT	p_val_adj
Mgp	5.74E-45	-0.34061	0.045	0.26	6.96E-41
Gm26917	5.18E-30	-0.43774	0.304	0.497	6.27E-26
B2m	1.06E-27	-0.2647	0.999	1	1.28E-23
Ccl5	3.97E-26	-0.38118	0.288	0.484	4.82E-22
Ifi27l2a	3.35E-22	-0.46208	0.942	0.982	4.06E-18
AY036118	1.23E-21	-0.37158	0.23	0.4	1.49E-17
Uba52	2.87E-19	-0.34234	0.739	0.812	3.47E-15
Ccl8	1.02E-16	-0.31664	0.117	0.251	1.23E-12
C1qb	4.52E-16	-0.43607	0.69	0.819	5.49E-12
C1qa	8.17E-16	-0.4778	0.608	0.749	9.91E-12
H2-Ab1	1.16E-15	-0.40508	0.974	0.986	1.41E-11
Srsf5	3.20E-15	-0.28675	0.874	0.916	3.88E-11
Psmb8	4.88E-15	-0.3058	0.923	0.939	5.91E-11
H2-Eb1	1.15E-14	-0.3704	0.907	0.957	1.39E-10
Cd74	8.58E-14	-0.37676	0.996	1	1.04E-09
H2-Aa	9.28E-14	-0.3882	0.95	0.963	1.12E-09
Gm42418	2.93E-12	-0.30393	0.438	0.536	3.55E-08
H2-T23	4.61E-12	-0.32658	0.419	0.516	5.59E-08
C1qc	1.45E-11	-0.46923	0.566	0.684	1.76E-07
Ly86	2.18E-11	-0.30756	0.752	0.807	2.65E-07
Plbd1	2.81E-11	-0.29819	0.818	0.849	3.40E-07
Ly6a	6.76E-11	-0.57326	0.296	0.415	8.20E-07
H2-DMa	9.10E-11	-0.32025	0.81	0.85	1.10E-06
AW112010	1.54E-10	-0.43684	0.445	0.549	1.87E-06
Aif1	3.16E-10	-0.33153	0.771	0.826	3.83E-06
Psmb9	4.15E-10	-0.26258	0.557	0.637	5.03E-06
H2-DMb1	6.79E-10	-0.37185	0.725	0.783	8.23E-06
Cxcl16	7.36E-08	-0.33742	0.695	0.755	0.000892
H2-Q7	1.75E-07	-0.27645	0.361	0.451	0.002123
Bst2	2.50E-07	-0.27031	0.742	0.785	0.003028

Cd83	6.23E-07	-0.28	0.758	0.794	0.007552
Cd72	2.76E-06	-0.3341	0.267	0.352	0.03349

Supplemental Table 8. Differentially upregulated genes in macrophage population isolated from atherosclerotic plaques of *Ldlr*$^{-/-}$ mice transplanted with WT or *Setdb2*^{GT} BM.

Gene	p_value	avg_logFC	Setdb2^{GT}	WT	p_val_adj
Cebpb	8.09E-53	1.030319	0.798	0.667	9.80E-49
Rps29	9.24E-33	0.325481	0.996	0.999	1.12E-28
Rpl38	7.64E-29	0.347442	0.978	0.979	9.26E-25
Rps28	3.60E-28	0.34411	0.977	0.975	4.36E-24
Hebp1	2.86E-24	0.505365	0.464	0.263	3.46E-20
Rps27	5.50E-24	0.309975	0.994	0.992	6.67E-20
S100a8	7.40E-23	0.358546	0.501	0.31	8.97E-19
Rps21	1.99E-22	0.296576	0.981	0.968	2.41E-18
Rpl36	4.23E-22	0.294402	0.973	0.981	5.13E-18
Rpl37a	1.02E-21	0.251495	1	0.996	1.24E-17
Rpl37	1.16E-21	0.272516	0.986	0.992	1.41E-17
Rpl41	1.26E-21	0.298641	1	0.999	1.53E-17
Hspa8	2.53E-18	0.31742	0.986	0.991	3.06E-14
Dynll1	8.09E-13	0.314833	0.922	0.921	9.81E-09
Basp1	2.09E-11	0.36082	0.684	0.592	2.53E-07
Apobec1	2.01E-10	0.393714	0.657	0.587	2.44E-06
2010107E04Rik	2.35E-10	0.285239	0.865	0.815	2.85E-06
Emp1	2.93E-10	0.369353	0.332	0.216	3.55E-06
Vim	4.89E-08	0.429591	0.92	0.918	0.000593
Cd9	4.77E-07	0.486396	0.711	0.638	0.005786
Spp1	5.86E-07	1.284653	0.607	0.53	0.007099
Clec4n	6.83E-07	0.408238	0.445	0.356	0.008283
Aldoa	7.66E-07	0.276612	0.873	0.861	0.009287
Myeov2	8.75E-07	0.29614	0.784	0.758	0.01061
Capg	1.83E-06	0.342404	0.733	0.679	0.022217
Tsc22d3	2.04E-06	0.29655	0.568	0.495	0.024758
Itgb5	3.96E-06	0.254233	0.688	0.649	0.048048

Supplemental Table 9. Differentially downregulated genes in macrophage population isolated from atherosclerotic plaques of *Ldlr*$^{-/-}$ mice transplanted with WT or *Setdb2*^{GT} BM.

Gene	p_value	avg_logFC	Setdb2^{GT}	WT	p_val_adj
AY036118	1.64E-24	-0.33125	0.206	0.412	1.99E-20
Mgp	3.84E-23	-0.31596	0.116	0.315	4.65E-19
Ly6a	3.05E-22	-0.66008	0.415	0.631	3.69E-18
Ly6e	4.82E-19	-0.45272	0.853	0.94	5.84E-15
Ccl8	6.92E-16	-0.29828	0.266	0.455	8.39E-12
Gm10116	2.74E-14	-0.27148	0.235	0.389	3.32E-10
Uba52	4.16E-14	-0.28582	0.666	0.768	5.04E-10
Psmb8	9.95E-14	-0.25287	0.871	0.931	1.21E-09
H2-Q7	1.18E-13	-0.38936	0.47	0.616	1.43E-09
AW112010	1.43E-13	-0.39122	0.621	0.756	1.74E-09
Ccl4	2.26E-13	-0.40663	0.688	0.804	2.74E-09
H2-T22	1.18E-12	-0.30574	0.433	0.585	1.43E-08
Psmb9	3.93E-11	-0.25256	0.591	0.719	4.76E-07
Bst2	4.65E-11	-0.30648	0.808	0.869	5.64E-07
Dek	1.39E-10	-0.36321	0.537	0.641	1.68E-06
Egr1	3.02E-10	-0.4495	0.379	0.506	3.66E-06
Nr4a1	5.74E-09	-0.27927	0.622	0.744	6.95E-05
H2afv	1.93E-08	-0.48721	0.494	0.609	0.000234
Ccl7	3.28E-08	-0.36559	0.402	0.536	0.000398
Nrp1	3.71E-08	-0.2646	0.425	0.53	0.00045
Ifitm3	5.39E-08	-0.33954	0.846	0.885	0.000653
Ifi27l2a	1.14E-07	-0.28439	0.846	0.913	0.001377
Irf1	2.41E-07	-0.27357	0.344	0.461	0.002918
Isg15	5.41E-07	-0.28528	0.38	0.493	0.006564
Id3	8.21E-07	-0.33396	0.415	0.514	0.009948

Supp Table 10. Clinical features of patient samples.

**Atherosclerosis**			**None-Mild**	**Mod-Severe**	***P* value**	
**Patient number**			*n* = 3	*n* = 7		
**Subject characteristics**	**Age**	**mean ± SD**	37.3 ± 25.3	58.9 ± 10.1	0.08	t-test
	**Sex**	***n*(%) male**	3 (100%)	4 (57.1%)	0.48	Fisher's exact test
	**Race**	***n* (%) Caucasian**	3 (100%)	6 (85.7%)	>0.99	Fisher's exact test
	**Ethnicity**	***n*(%) Hispanic**	1 (33.3%)	3 (42.9%)	>0.99	Fisher's exact test
**Subject operation**	**Transplant donor or recipient**	***n*(%) donors**	2 (66.7%)	6 (85.7%)	>0.99	Fisher's exact test
**History of atherosclerosis**	**Coronary artery disease**	***n*(%)**	0 (0)%)	2 (28.6%)	>0.99	Fisher's exact test
	**Cerebrovascular disease**	***n*(%)**	0 (0)%)	1 (14.3%)	>0.99	Fisher's exact test
	**Peripheral vascular disease**	***n*(%)**	0 (0)%)	0 (0)%)	>0.99	Fisher's exact test
**Risk factors for atherosclerosis**	**Hypertension**	***n*(%)**	1 (33.3%)	4 (57.1%)	>0.99	Fisher's exact test
	**Hyperlipidemia**	***n*(%)**	0 (0)%)	1 (14.3%)	>0.99	Fisher's exact test
	**Diabetes**	***n*(%)**	0 (0)%)	1 (14.3%)	>0.99	Fisher's exact test
	**Smoker**	***n*(%)**	1 (33.3%)	5 (71.4%)	0.50	Fisher's exact test
